# The Physicochemical and Antimicrobial Properties of Silver/Gold Nanoparticles Obtained by “Green Synthesis” from Willow Bark and Their Formulations as Potential Innovative Pharmaceutical Substances

**DOI:** 10.3390/ph16010048

**Published:** 2022-12-29

**Authors:** Roxana Colette Sandulovici, Mihailescu Carmen-Marinela, Alexandru Grigoroiu, Carmen Aura Moldovan, Mihaela Savin, Viorel Ordeanu, Sorina Nicoleta Voicu, Daniel Cord, Gabriela Mariana Costache, Mona Luciana Galatanu, Mariana Popescu, Iulian Sarbu, Erand Mati, Lucia Elena Ionescu, Răzvan Neagu, Vasilica Ţucureanu, Rîmbu Mirela Claudia, Iuliana Mihalache, Cosmin Romanitan, Alice Piperea-Sianu, Adina Boldeiu, Oana Brincoveanu, Carmen Elisabeta Manea, Bogdan Firtat, George Stelian Muscalu, David Dragomir

**Affiliations:** 1 Pharmacy Faculty, “Titu Maiorescu” University, 16 Sincai, 040314 Bucharest, Romania; 2 National Institute for Research and Development in Microtechnologies (IMT Bucharest), 126A Erou Iancu Nicolae Street, 72996 Bucharest, Romania; 3 “Cantacuzino” National Institute for Medical-Military Research-Development, 050096 Bucharest, Romania; 4 Department of Biochemistry and Molecular Biology, Faculty of Biology, University of Bucharest, 91–95 Splaiul Independentei, 050095 Bucharest, Romania; 5 Faculty of Pharmacy, “Carol Davila” University of Medicine and Pharmacy, 050474 Bucharest, Romania; 6 Independent Researcher, 023831 Bucharest, Romania; 7 Horia Hulubei National Institute for R&D in Physics and Nuclear Engineering, 30 Reactorului Street, 077125 Magurele, Romania; 8 Faculty of Mechanical Engineering and Mechatronics, University Politehnica of Bucharest, Splaiul Independentei 313, 060042 Bucharest, Romania

**Keywords:** silver nanoparticles, polyphenols, willow bark, *Salix alba*, antimicrobial effect, *Pseudomonas aeruginosa*, pharmaceutical formulations

## Abstract

Green chemistry is a pharmaceutical industry tool, which, when implemented correctly, can lead to a minimization in resource consumption and waste. An aqueous extract of *Salix alba* L. was employed for the efficient and rapid synthesis of silver/gold particle nanostructures via an inexpensive, nontoxic and eco-friendly procedure. The nanoparticles were physicochemically characterized using ultraviolet–visible spectroscopy (UV–Vis), Fourier transform infrared spectroscopy (FT-IR), dynamic light scattering (DLS), X-ray diffraction (XRD) and scanning electron microscopy (SEM), with the best stability of up to one year in the solution obtained for silver nanoparticles without any chemical additives. A comparison of the antimicrobial effect of silver/gold nanoparticles and their formulations (hydrogels, ointments, aqueous solutions) showed that both metallic nanoparticles have antibacterial and antibiofilm effects, with silver-based hydrogels having particularly high antibiofilm efficiency. The highest antibacterial and antibiofilm efficacies were obtained against *Pseudomonas aeruginosa* when using silver nanoparticle hydrogels, with antibiofilm efficacies of over 75% registered. The hydrogels incorporating green nanoparticles displayed a 200% increased bacterial efficiency when compared to the controls and their components. All silver nanoparticle formulations were ecologically obtained by “green synthesis” and were shown to have an antimicrobial effect or potential as keratinocyte-acting pharmaceutical substances for ameliorating infectious psoriasis wounds.

## 1. Introduction

Over the past decade, it has been demonstrated that many biological systems, including plants and algae [1], can transform inorganic metal ions into metal nanoparticles (NPs) via the reductive capacities of the proteins and metabolites present in these organisms. Apart from their inherent environmentally friendly characteristics, plant-produced NPs have been shown to offer significant industrial advantages, such as cost-effectiveness and an ease of translation to large scale production [2]. This is in contrast to chemically/physically synthesized NPs, which can be especially expensive and can affect the environment [3,4,5,6,7]. That is because chemical syntheses, such as Turchevich and Tollens, use non-polar solvents, chemical reducing agents (e.g., sodium borohydride, sodium citrate and CTT) and/or chemical additives for their stabilization in solutions [8,9]. Thus, studies towards “green syntheses” have recently taken precedence. “Green synthesis” makes use of metal salts and reducing, additive and stabilizing compounds obtained from plants, microorganisms, bacteria and fungi, which leads to a prevalence of naturally occurring biofactories for nanoparticles. Microorganisms such as bacteria, fungi, yeasts and viruses have an innate potential for the production of metal nanoparticles either intra- or extracellularly. NP biosynthesis is not limited to natural biofactories, with techniques that employ phytochemical compounds from parts of plants (e.g., flowers, leaves and stems) covering a wide range of metabolites and being more profitable [10,11]. An additional advantage of nanomaterial “green synthesis” is that the natural extracts counteract the effects of metal nanoparticles, thus, making the nanomaterials stable and safe. It has also been demonstrated that NPs synthesized using plant extracts are more biocompatible and biologically active than those synthesized via chemical methods [12,13,14,15]. Hence, silver NPs, obtained via plant metabolites enriched with polyphenols and formulated with natural polymers, could lead to obtaining highly therapeutic effects compared to their individual components. In a recent study, a wound dressing material based on the combination of gelatin, chitosan and silver nanoparticles was developed to significantly reduce injury infection rates and to accelerate local vascularization and scarring [16]. In vitro studies on polyphenol-enriched nanoparticles from *Cornus mas* have also shown their contribution to the suppression of NF-κB activation in macrophages [17]. This effect appears to be amplified in vivo, such as in inflamed psoriasis skin, inhibiting the production of pro-inflammatory cytokines and with a causal role in psoriasis pathogenesis (TNF-α and IL-12) [18,19]. Although a clear understanding of the phytochemicals involved in the “green synthesis” of NPs would help us devise new strategies to control physicochemical properties, such as NP size and shape, it remains challenging due to the complexity of plant extracts. However, the potential of phytochemical compounds is yet to be fully utilized for the synthesis of metal nanoparticles on a large scale in the pharmaceutical and related industries [20,21,22,23]. The white willow tree (WT), in particular, is known for containing polyphenols in addition to salicin, which is conducive to moisture retention and photodamage protection while also exhibiting anti-inflammatory and antibacterial properties [24,25]. While white willow has primarily been employed for synthesizing gold nanoparticles from its leaves, instances of synthesizing silver NPs (Ag-NPs) from its bark have also been successful [26]. Such naturally produced Ag-NPs have been widely used in formulations for contact lens hydrogels to reduce microbial infection, with their efficiency tested on bacteria isolated from dental plaque [27]. This is due to the inherent antimicrobial effect of silver and the demonstrated efficiency of Ag-NPs as antimicrobial agents in pharmaceutical applications for wound- and skin-related infections [22,23,24,25,26,27,28,29,30]. There are also few existing marketed nano-pharmaceutical formulations of Ag-NPs synthesized from plants [31]. When bringing Ag-NP products to the market as either food supplements or medicine, it is imperative that they are appropriately tested for toxicity and the size of colloidal silver, as 1 nm colloidal silver can be safely passed through the body without deposition, which corresponds to those NPs produced naturally. Thus, physicochemical characterization (particle size, shape, structure, chemistry, crystallography, etc.), as well as tests for toxicity and antimicrobial activity, are essential to qualify nanoparticles for use in pharmaceutical applications [32,33]. Even when antimicrobial activity exists for nanoparticles, their introduction in different formulations (gels and creams) can potentiate this activity or cancel it. Formulation versions with both silver NPs (SAgNPs-WT) and gold NPs (SAuNPs-WT) extracted from white willow bark were characterized in colloidal solutions using UV–Vis, DLS, zeta potential, attenuation total reflection (ATR), FT-IR and SEM, with the aim of determining their physicochemical properties. Of the two, the SAgNPs-WT showed better physicochemical properties in solution and stability over time. Two main pharmaceutical formulations using SAgNPs-WT were considered in this article: a hydrogel based on chitosan and gelatin and an ointment based on lanolin and Vaseline. Due to the instability of SAuNPs-WT in these two formulations, gold nanoparticles were only characterized and compared in aqueous solutions. With the solutions being tested for targeting psoriasis and half the wounds resulting from psoriasis being caused by bacterial agents, the antimicrobial activity of the formulations was determined against three bacterial cultures: *Escherichia coli* (*E.c*), *Staphylococcus aureus* (*S.a*) and *Pseudomonas aeruginosa* (*P.a*), with various grades of success on all three cultures. Meanwhile, the anti-psoriatic effects of silver nanoparticles were also evaluated in vitro on psoriasis-like keratinocytes obtained from HaCaT cell lines. Green-synthesized silver nanoparticles with the required physicochemical properties for integration in novel pharmaceutical formulations with a potential effect on the treatment of wounds such as those arising from psoriasis infections are, thus, presented and characterized in this work while being compared to their gold homologues.

## 2. Results and Discussion

### 2.1. Synthesis of SAgNPs-WT

**Figure 1:**
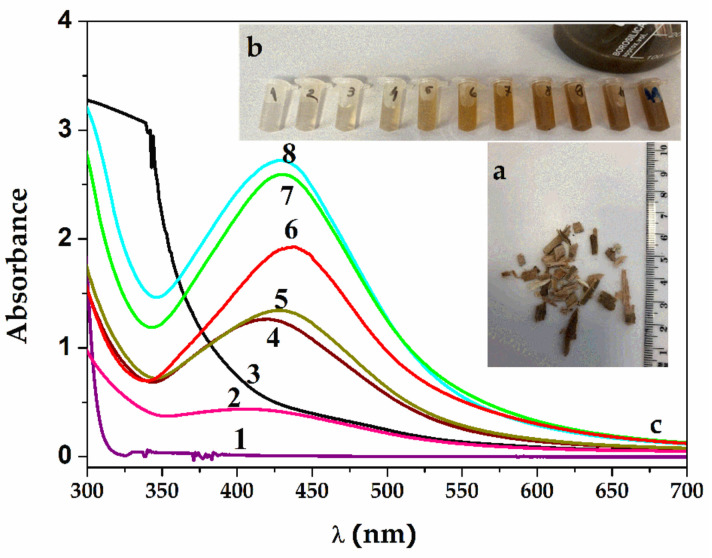
(**a**) *Salix alba L.* bark was collected in Dâmboviţa County, Romania, from willow trees. (**b**) Color change of the solution from yellow to dark brown. (**c**) UV–Vis spectra of SAgNP-WT samples obtained by reduction in time with willow aqueous extract: 1—AgNO_3_ 0.5 mM; 2—SAgNPs-WT 1S (after 5 min); 3—willow extract; 4—SAgNPs-WT 4S (after 10 min); 5—SAgNPs-WT 5S; 6—SAgNPs-WT 7S; 7—SAgNPs-WT 9S; and 8—SAgNPs-WT 10S.

The formation of Ag-NPs during biosynthesis was confirmed by the color change of the solution from yellow to dark brown after adding 18 mL of *Salix alba (*Figure 1a shows the willow tree bark) extract to 80 mL of AgNO_3_ (0.5 mM) after a 30 min reaction at 60 °C, as shown in Figure 1b. A unique property of spherical Ag-NPs is that the peak wavelengths in surface plasma resonance (SPR) can be changed from 400 nm (violet light) to 530 nm (green light) by changing the particle size and refractive index near the particle surface [31,34]. Thus, to determine the Ag-NP sizes, UV–Vis spectrometry was used to record their wavelengths and correlate them with the size of the nanoparticles obtained after their synthesis. UV–Vis absorption and the maximum wavelength of the Ag-NPs are given by the size, shape and environment in which they are dispersed, the density of free electrons and their interactions with other chemical compounds in the environment [28]. The phytochemical compounds in the *Salix alba* bark extract were silver reducers and stabilizers, and the addition of repeated volumes of the extract led to an increase in the concentration of silver nanoparticles.

The spectra from Figure 1c represent the appearance of an initial absorption maximum after 5 min of reaction at a wavelength of 409 nm (spectra 2), manifesting as a widening band and, thus, showing a polydispersity of the silver nanoparticles formed. This band narrows and reaches a wavelength of 420 nm after 10 min (spectra 4). After 15 min of continued reaction, the recorded absorption maximum shifts to 436 nm, and on reaching 30 min, the absorption maximum bands stabilize at 434 nm (curves 5–8). The few existing studies show an absorption maxima of 440 nm [20] and 447 nm [35] for the biosynthesis of silver nanoparticles from white willow. The size is directly related to the λmax of SPRs, with diameters of 20 to 40 nm being observed for the resulting SAgNPs-WT.

### 2.2. Synthesis of SauNPs-WT

For the synthesis of SauNPs-WT, the protocol was the same as for SAgNPs-WT, with the ionic salt used to reduce the gold being 0.1 mM HauCl_4_x3H_2_0.

**Figure 2:**
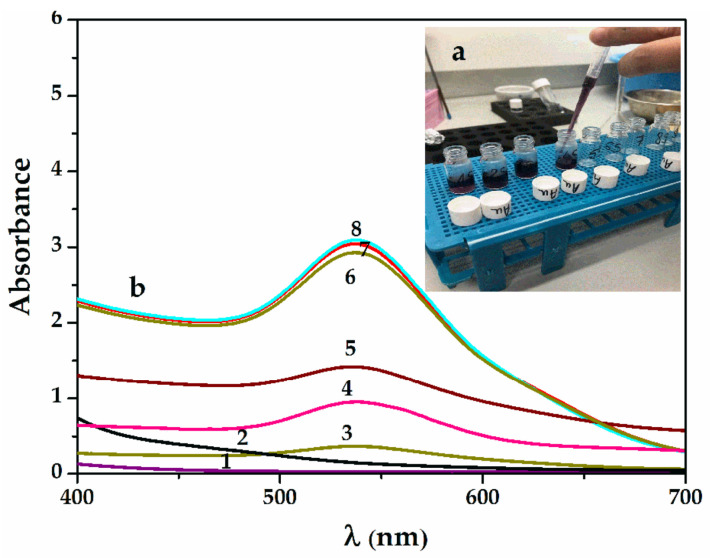
(**a**) Samples collected during gold bioreduction with the appearance of the purple–violet final color; (**b**) UV–Vis spectra of SauNP-WT samples obtained by reduction in time with willow aqueous extract: 1—0.1 mM HauCl_4_x3H_2_0; 2—willow extract (after 5 min); 3—SauNPs-WT 1S; 4—SauNPs-WT 4S; 5—SauNPs-WT 5S; 6—SauNPs-WT 7S; 7—SauNPs-WT 9S; and 8—SauNPs-WT 10S.

Figure 2a shows the UV–Vis spectra during the reaction, with the appearance of the purple–violet final color shown in Figure 2b. The reaction was rapid, with a wide band of maximum absorption formed at 535 nm in the first 5 min and only a narrowing of the band observed after 30 min at a rate of 1:5.7 (*v:v*) for the aqueous willow extract and chloroauric solution. This may explain the lower polydispersity at the end of the reaction. The wavelength shows a slightly smaller particle size than in the case of silver nanoparticles at around 15 to 30 nm. The SEM images further confirm the sizes of the Sag/AuNPs-WT.

### 2.3. Physicochemical Characterization of the Nanoparticles

#### 2.3.1. X-ray Diffraction Analysis

**Figure 3:**
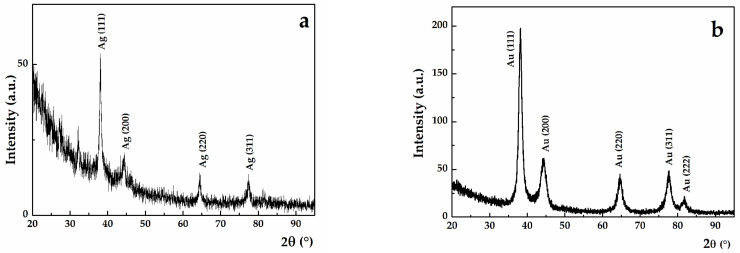
Grazing incidence X-ray diffraction (GIXRD) pattern for (**a**) SAgNPs-WT and (**b**) SAuNPs-WT. The peaks were indexed according to the ICDD database.

The experimental peaks were located at 38.25°, 44.63°, 64.80°, 77.79° and 81.90°, which correspond to the (111), (200), (220), (311) and (222) Bragg reflections for SAuNPs-WT. Meanwhile, for SAgNPs-WT, the experimental peaks were located at 38.03°, 44.35°, 64.37° and 77.37°. These experimental peaks correspond to gold and cubic silver, respectively, as indexed by the ICDD database with cards no. 001-1172 and 001-1164. Qualitatively, it can be observed that the full width half maximum (FWHM) of the (111) diffraction peak decreases from 1.54° for AuNPs to 0.55° for SAgNPs-WT (Figure 3a,b). From a quantitative point of view, the decrease in the peak width is correlated with an increase in the mean crystallite size, according to the Debye–Scherrer equation. A size of 5.67 nm was found for Au-NPs, while Ag-NPs exhibited larger mean crystallite sizes (16.12 nm). The presence of XRD peaks and mean crystallite sizes of several nm indicate that the synthesized NPs were nanocrystalline.

#### 2.3.2. Scanning Electron Microscopy Imaging of the Nanoparticles

**Figure 4:**
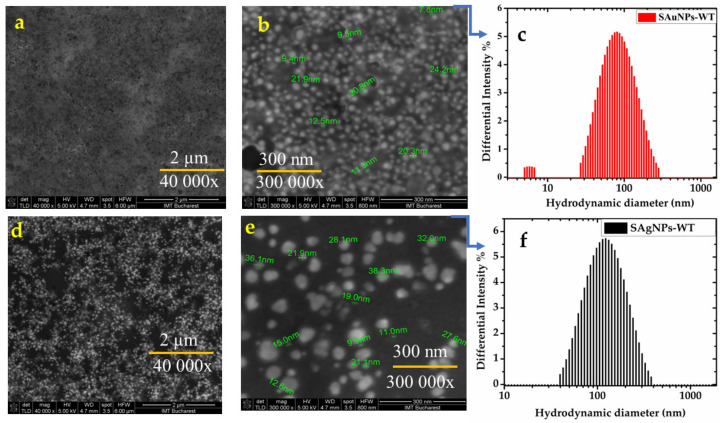
SEM images at different magnitudes and hydrodynamic diameters determined by DLS: (**a**)—SAuNPs-WT (40,000×); (**b**)—SAuNPs-WT (300,000×); (**c**)—DLS of SAuNPs-WT; (**d**)—SAgNPs-WT (40,000×); (**e**)—SAgNPs-WT (300,000×); and (**f**)—DLS of SAgNPs-WT.

The recorded SEM images for SAu/AgNPs-WT are shown in Figure 4a,b for SAuNPs-WT and Figure 4d,e for SAgNPs-WT, with spherical gold/silver nanoparticles with an average size distribution of 8–25 nm (average size, 19.5 ± 0.7 nm) observed for SAuNPs-WT, while those for AgNPs-WT were larger at 10–38 nm (average size, 21.5 ± 0.7 nm). Histograms of the Sau/AgNPs-WT size distributions were fitted by Gaussian functions and are presented in Figure S1a,b. The obtained values are in agreement with previously collected data for *Salix alba* nanoparticles (gold/silver) extracted from its leaves and bark, respectively [27,36,37].

#### 2.3.3. Dynamic Light Scattering and Zeta Potential for the Polydispersity of the Nanoparticles

Hydrodynamic and electrophoretic light scattering (DLS/ELS) measurements were conducted to obtain information regarding the hydrodynamic diameter (dh) and zeta potential (ζ) to predict the long-term stability of the Sag/AuNP-WT colloidal solutions obtained after the bioreduction in the aqueous *Salix alba* extract. For example, particles with zeta potentials greater than ±60 mV have excellent stability, whereas particles with zeta values between −10 mV and +10 mV will experience rapid agglomeration unless sterically protected. The sign and magnitude of the zeta potential can be used as a secondary metric to determine surface chemical changes. A polydispersity index (PDI)—a measure of particle size homogeneity—smaller than 0.3 is expected for homogeneous populations of green nanoparticles, with the potential of NPs for different pharmaceutical formulations being linked to their PDI. PDI values of 0.2 and lower are considered acceptable in practice for single nanoparticles or for particles coated with biological compounds or polymeric materials. Meanwhile, the sizes determined by DLS can differ when compared to those obtained by SEM measurements due to their different operating principles, with DLS also including the surface coating in its determination of the particle size. Therefore, dh values of 71 nm were obtained for SAuNPs-WT, while for SAgNPs-WT, values of 106.9 nm were observed, as can be seen in Figure 4c,f. The zeta potential shows information about the surface charge of nanoparticles, with SAgNPs-WT presenting a higher negative potential (−84.45 mV) at 0.193 PDI, while SAuNPs-WT had low values of negative potential at −4 mV and 0.267 PDI. Accordingly, the SAgNP-WT nanoparticles were covered by anionic surface charges, which were sufficient for stabilizing the NPs in the colloidal solution after reaction. However, SAuNPs-WT are not as stable as silver NPs, presenting more polydispersity and two distributions of the measured population. While the PDI for SAuNPs-WT still makes them acceptable, the low value of their ζ potential intuits a tendency to flocculate over time, thus, making them unsuitable for use in future pharmaceutical formulations, as they cannot be covered by biocompatible polymers or chemical capping agents for stabilization. Hence, only SAgNPs-WT, with their surrounding hydrophilic functional groups, are colloid-stable in an aqueous medium.

#### 2.3.4. UV–Vis Characterization for Temporal Stability and the Influence of pH

The stability of the SAgNPs-WT was also evaluated spectrophotometrically after one year following synthesis. The UV–Vis spectra presented in Figure S2 show no notable modifications in the maximum absorption band after storing the solutions for one year at room temperature. The possibility of using the SAgNPs-WT in different pH media was also evaluated through UV–Vis characterization, with Figure S3 showing the characteristic spectra of colloidal solutions with SAgNPs-WT modified by adjusting the pH with 1 M of NaOH or 1 M of HCl solutions. We can observe their stability in a pH range between 2 and 8; the bands start to widen with increasing pH values. The solutions showed very good stability in acidic environments but also did not show major changes in the initial wavelength when they had a slightly basic pH.

#### 2.3.5. FT-IR Spectroscopy Analysis

**Figure 5:**
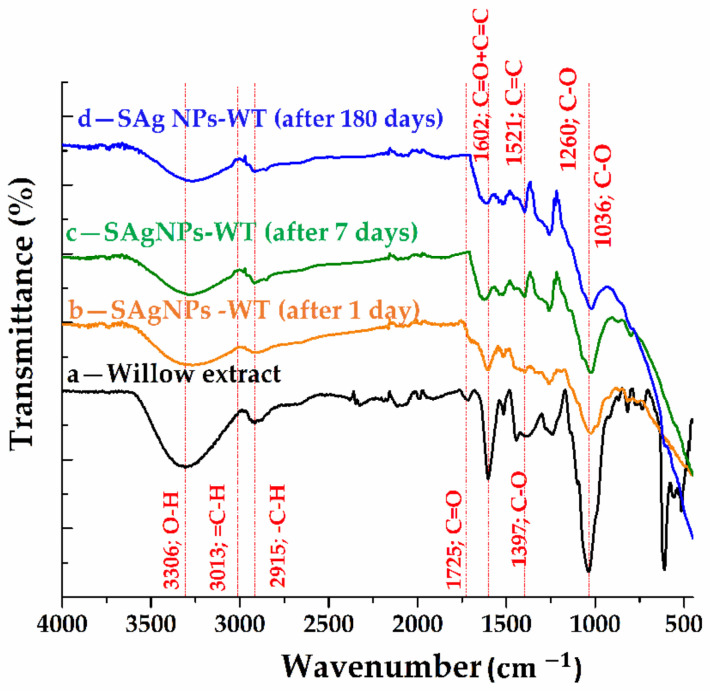
ATR-FTIR spectra for (a) willow extract and silver nanoparticles, (b) 1 day, (c) 7 days and (d) 180 days from synthesis.

The ATR-FTIR spectra recorded for the willow extract and the samples of SAgNPs-WT recorded at 1 day, 7 days and 180 days, respectively, after the synthesis, are presented in Figure 5.

Table S1 shows the possible assignments of the absorption bands for the four samples. With respect to chemical composition, willow extract is a complex compound containing hexose sugars (fructose, glucose, mannose and xylose), tannins (polyphenolic molecules) and mineral substances in different proportions. In Figure 5(d), we can see a broad band centered at 3306$\;{\mathrm{cm}}^{-1}$, which can be assigned to the symmetric vibration mode of the O-H bonds and peaks of reduced intensity in the 3100–2900 ${\mathrm{cm}}^{-1}$ range, which can be attributed to =C-H bonds (aromatic compounds’ stretching vibration) and -C-H (aliphatic bonds’ stretching vibration). The existence of phenolic aromatic groups and alcoholic aliphatic groups from both hexoses and tannins can be confirmed by the existence and correlation of these bands. The peak centered at 1725$\;{\mathrm{cm}}^{-1}$ can be attributed to the symmetric vibration mode of the bonds involved in the C=O from the non-conjugated carbonyl groups (ketones or aldehydes). The conjugation of the carbonyl groups with the aromatic ring is supported by the appearance of a 1600 ${\mathrm{cm}}^{-1}$ band. Moreover, the existing aromatic structure is supported by the appearance of the band at 1516 ${\mathrm{cm}}^{-1}$.

The 1395 ${\mathrm{cm}}^{-1}$ band confirms the existence of phenolic compounds. Meanwhile, the 1240 ${\mathrm{cm}}^{-1}$ band can be attributed to the vibration mode of C–O bonds involved in the guaiacol rings or syringyl rings from tannins, while the band at 1036 ${\mathrm{cm}}^{-1}$ can be associated to the mode of deformation of C–O bonds from alcohols or aliphatic ethers. Comparing the synthesis sample spectra of silver nanoparticles (Figure 5b–d) with the willow extract spectrum, a change in the spectrum below 700 ${\mathrm{cm}}^{-1}$ was observed, thus, confirming the formation of Ag nanoparticles to which the willow groups are anchored. The anchoring of organic molecules to silver nanoparticles is confirmed by the presence of bands in the 4000–700 region. These bands are slightly shifted compared to the spectral bands for the willow sample, and it can be said that the silver nanoparticles present in the FT-IR spectrum exhibit vibrations characteristic of the alcohols and phenolic groups from the willow extract anchored by the nanoparticles. The peak reduction after 1 day of synthesis at about 3300, 2915 and 1700 ${\mathrm{cm}}^{-1}$, simultaneously with the disappearance of the band at 1700 and 1440 ${\mathrm{cm}}^{-1}$ after 7 days, confirms the involvement of hydroxyl and carbonyl groups in the reduction in salts and the stabilization of silver nanoparticles. For the samples recorded at the 180-day mark, the stability of the synthesized nanomaterials is confirmed. Thus, it can be said that willow acts as both a reducing and a capping agent [24,25,29,38].

Taking into account all the characterizations previously presented, the green SAgNPs-WT show increased stability over time and may be used alone in different pharmaceutical formulations such as hydrogels, ointments, aqueous/alcoholic solutions, gels and soaps.

#### 2.3.6. Florescence Imaging of the Silver Nanoparticles

**Figure 6:**
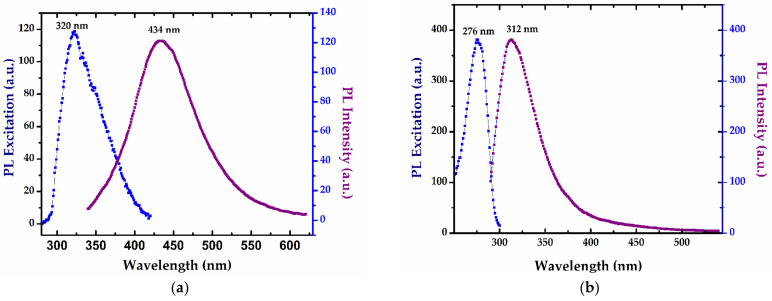

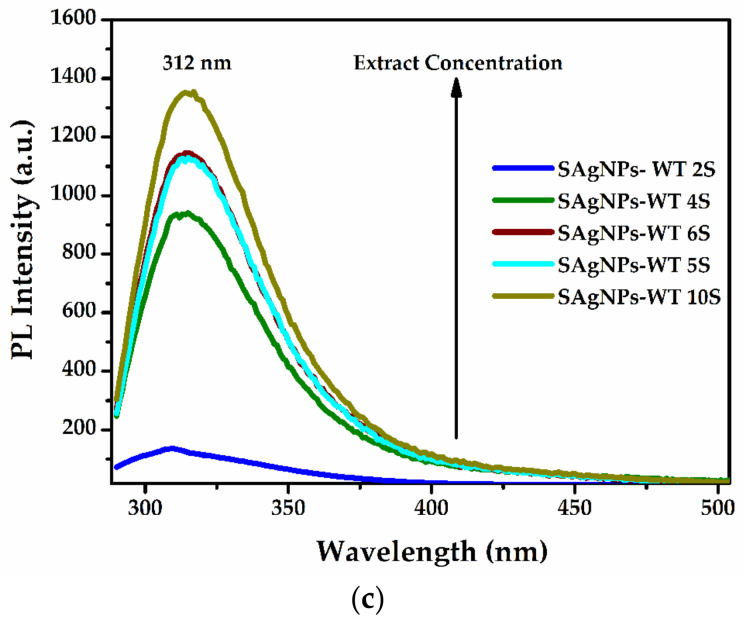
Photoluminescence excitation (PLE) and emission (PL) spectra recorded for the SAgNPs-WT colloidal dispersion at (**a**) 320 nm excitation; (**b**) band characteristics of tyrosine at λex/λem = 276/312 nm; and (**c**) increases in the band of tyrosine (312 nm) with the concentration of the extract during biosynthesis.

Photoluminescence excitation (PLE) and emission (PL) spectra recorded for the SAgNPs-WT colloidal dispersion are shown in Figure 6a–c. SAgNPs-WT prepared by the “green synthesis” method show PL emission in the visible range, with a maximum at 434 nm when excited at 320 nm corresponding to the interbond radiative recombination of sp electrons to d holes intensified by the strong SPR excitation of silver nanoparticles (Figure 6a) [39]. The presence of small quantities of amino acids was evidenced by the emission spectrum obtained at 280 nm, with a maximum at 312 nm, and the excitation spectrum with a maximum at 276 nm, which are the characteristic bands of tyrosine (Figure 6b).

A slight red shift was identified when compared with free tyrosine whose fluorescence is located at λex/λem = 274/310 nm. Furthermore, the PL emission of eight samples of SAgNPs-WT extracted during synthesis at 3–5 min intervals were analyzed and are presented in Figure 6c. The intensity of the 312 nm PL peak shows that the tyrosine concentration significantly increases as a function of reaction time with the increase in extract quantity added during the synthesis. This can be another confirmation of the existence of polyphenolic compounds that exhibit fluorescence and whose fluorescence increases with the formation of silver nanoparticles.

### 2.4. Physicochemical Characterization of the Hydrogels with SAgNPs-WT

**Figure 7:**
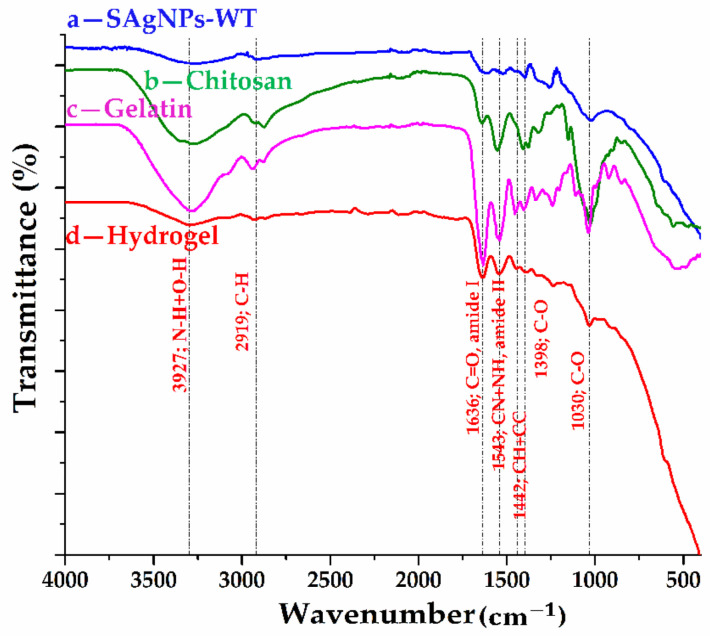
ATR-FTIR spectra for (a) silver nanoparticles from willow tree; (b) chitosan; (c) gelatin; and (d) hydrogel with silver nanoparticles.

The main structural composition of the raw materials (chitosan, gelatin and silver nanoparticles) and of the hydrogels with silver nanoparticle contents, obtained according to the previously presented biogenic protocol, can be deduced from the FT-IR spectra shown in Figure 7 and the possible assignment of the spectral bands in Table S2.

Depending on the process parameters, it was found that hydrogels based on gelatin and chitosan can lead to different types of interactions (electrostatic, hydrogen bonds, Van der Waals, ionic or covalent), with the formation of a three-dimensional network due to the gelatin. In the spectrum of the hydrogel presented in Figure 7d, a mixture of bands characteristic of not only the amine groups of chitosan but also of the carboxyl groups of gelatin can be observed. In the spectra of chitosan and gelatin, a series of similar bands can be observed, such as the broad band between 3600 ${\mathrm{cm}}^{-1}$ and 3000 ${\mathrm{cm}}^{-1}$, due to the overlap of the O-H and N-H bonds from the amide A group; absorption bands that can be attributed to the vibration mode of the C=O bonds in amide I (1634 ${\mathrm{cm}}^{-1}$ and 1638 ${\mathrm{cm}}^{-1}$, respectively) and to the coupling between the CN and NH bonds in amide II (1549 ${\mathrm{cm}}^{-1}$ and 1553 ${\mathrm{cm}}^{-1}$, respectively) and amide III (1280–1240 ${\mathrm{cm}}^{-1}$); and bands associated with the vibration mode of C-H bonds (mainly the amide B bands in the field 3100–2800 ${\mathrm{cm}}^{-1}$) and C–O from both carboxylic or ethoxy groups (1410–1000 ${\mathrm{cm}}^{-1}$). The FT-IR spectrum of the metallic nanoparticles was discussed above, confirming the existence of some groups from the willow extract on the Ag NP surface. Compared to the spectra of the raw materials, in the case of the hydrogel with silver nanoparticle contents, a slight shift of the peak associated with the bonds from amide A (up to 3297 ${\mathrm{cm}}^{-1}$) can be observed as a result of the appearance of hydrogen bonds. The formation of a complex network involving hydrogen bonds in the hydrogel structure can also be confirmed by the slight shift of the amide bands I (up to 1636 ${\mathrm{cm}}^{-1}$), II (up to 1543 ${\mathrm{cm}}^{-1}$) and III (up to 1237 ${\mathrm{cm}}^{-1}$) in the hydrogel spectrum. The network distortion can also be confirmed by changing the position of the peak’s characteristic of C-H bonds (up to 3077, 2919 and 2873 ${\mathrm{cm}}^{-1}$). The interaction between the silver nanoparticles and the components of the hydrogel is supported by the shift of the bands that can be associated with the C–O bonds from the carboxyl group (up to 1398, 1197, 1156 and 1030 ${\mathrm{cm}}^{-1}$), as well as by the lack of spectral bands below 900 ${\mathrm{cm}}^{-1}$ [37,40,41,42,43,44].

Hydrogel samples for SEM were prepared as follows: 0.001 mg of hydrogel was deposited on a silicon surface and was dehydrated by heating for 24 h at 60 degrees. Gelatin, chitosan and aqueous willow extract samples with SAgNPs-WT were also dehydrated for 24 h on a silicon wafer. The main advantage of this hydrogel is that a uniformity of SAgNPs-WT in its structure was obtained without the addition of an additional stabilizer. This can be confirmed in an SEM image from Figure S4, where nanoparticles uniformly distributed between networks of polymer chains can be seen.

### 2.5. Antimicrobial Results

A number of substances and formulations without/or in combination with metal nanoparticles obtained ecologically by “green synthesis” were tested as potential antimicrobial substances: hydrogels and ointments with SAgNPs-WT, metallic green nanoparticle Sag/AuNPs-WT and controls, such as different dilutions of AgNO3, chitosan and glycerin. A diagram of the testing plate and the employed samples for the antimicrobial tests is presented in Supplementary Figure S5.

#### 2.5.1. SAg/AuNPs-WT

- Diffusimetric method

To avoid the overlap of the test substance absorbance with the absorbance of the bacterial multiplication, the diffusimetric antibiogram technique was employed with different concentrations and quantities of the test substance solutions. For analysis, the obtained inhibition zones (ZOI), measured in millimeters, were recorded, with the two different diameters (minimum and maximum) being compared for correlation with the microplate test method.

Thus, it was observed that the positive control (AgNO_3_ 1N) had an antibacterial effect on all tested bacteria (Table S3). Meanwhile, SAgNPs-WT 10S and hydrogel A had an antibacterial effect on *P.a,* while the ointment with SAgNPs-WT 10S and Tween 20 had an effect on *S.a* and *P.a*, as presented in Table 1.

SAgNP-WT 10S nanoparticles (those obtained after the end of biosynthesis) were tested against other innovative antimicrobial substances on the three representative bacterial species. Some of these (gelatin 2.5% in water; chitosan + gelatin; hydrogel with chitosan + gelatin SAgNPs-WT; and sterile hydrogel with chitosan + SAgNPs-WT) present a reduced diffusion in the culture medium, which led to the impossibility of correctly interpreting a possible antibacterial effect. SAgNPs-WT obtained by “green synthesis” from willow bark were tested compared to different pharmaceutical forms, with and without active substances, to highlight the possible positive and negative control effect, with an antibacterial effect observed on all three tested bacteria, as shown in Table 2.

- Antibacterial and antibiofilm effect

SAg/AuNPs-WT

As samples marked 1 to 10 S are taken sequentially during the synthesis, it is possible for the variation in the results regarding the bacterial activity to differ due to the varying particle sizes resulting from their different times of synthesis. It was observed that the nanoparticles had a polydispersity at the beginning of the synthesis with a variation in the wavelength from 430 nm to 436 nm, with it stabilizing at 434 nm. The lower wavelength at the beginning may be due to the formation of small nanoparticles that aggregate, with the antibacterial effect being lower, as a higher contact area with the bacteria results in increased antibacterial activity. Additionally, as can be seen from the FT-IR measurement, aromatic or polyphenolic compounds are prone to surround the nanoparticles, thus, enhancing the interaction of SAg/AuNPs-WT with bacteria via electrostatic attraction, with the polar heads of the lipids present in bacterial membranes favoring the insertion of nanoparticles into them [45,46,47].

**Figure 8:**
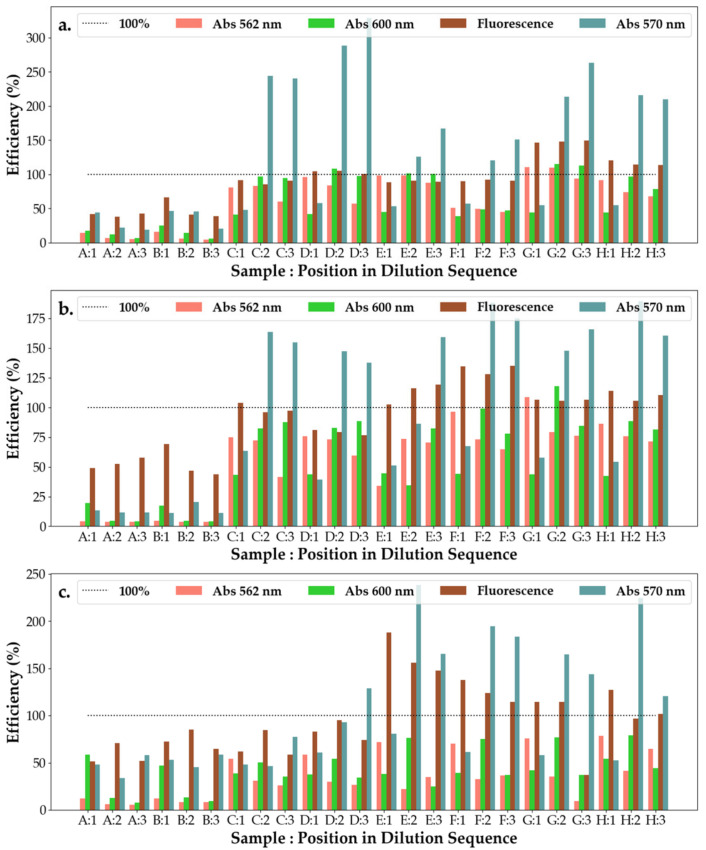
Efficiency of the antibacterial response (η) of the SAg/AuNP-WT samples at three levels of dilution tested against (**a**) *Escherichia coli*, (**b**) *Pseudomonas aeruginosa* and (**c**) *Staphylococcus aureus*. Samples A–H represent the following solutions tested against the bacteria: A—SAuNPs-WT 1S; B—SAgNPs-WT 1S; C—*Salix Alba* extract; D—*Salix Alba* extract; E—SAuNPs-WT 7S; F—SAuNPs-WT 10S; G—SAuNPs-WT 7S; and H—SAuNPs-WT 10S, as also shown in Figure S5.

From Figure 8, it can be observed that SAg/AuNP-WT solutions collected at the start of the synthesis (rows A and B) have the worst efficiency response, with their response remaining unchanged on subsequent dilutions and across bacteria. This points to solutions A and B being either highly inefficient or control substances with little interaction with the bacteria or effect on bacterial efficiency. This can be explained by the low concentration of extract added to the base solution being insufficient to start the formation of nanoparticles. With dilutions exhibiting a strong change in efficiency at 600 nm and a slighter change at 570 nm, this suggests that the nanoparticles are particularly sensitive to light at 600 nm. For *E.c* and *P.a*, the efficiency of the solutions increases with dilution (at 600 nm), whereas for *S.a*, the nanoparticle solutions appear to have only a small effect on the change in efficiency with it varying around 50% but displaying a small decreasing trend.

**Figure 9:**
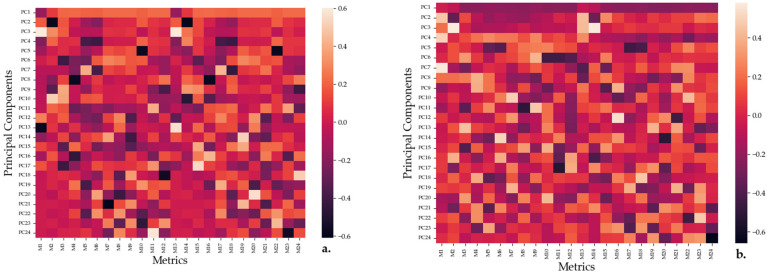
Feature maps representing the correlation between the information explained by the PCs approximated by PCA and the metrics/features collected while interrogating the SAu/AgNP-WT samples. A diagram of how each set of 12 features for a single wavelength is collected is given in Figure S5. (**a**) Feature map for the single wavelength measurements with M1 to M3 representing the blank, positive blank and negative blank responses and M4 to M12 representing a sequence of three repeats of each dilution measurement when the samples were interrogated with light at 562 nm. Similarly, M13–M24 express the absorbances of the samples when measured using 600 nm light. (**b**) Feature map for the multi-wavelength measurements with M1 to M3 representing the blank, positive blank and negative blank responses and M4 to M12 representing a sequence of three repeats of each dilution measurement when the samples were interrogated under fluorescence conditions. Similarly, M13–M24 express the absorbances of the samples when measured with light centered around 570 nm.

PCA components were mapped until a component explained less than 5% of the variation in data, with the 2D representations of the feature space being shown in Figure S6a for the fluorescent and 570 nm measurements and in Figure S6b for the 562 nm and 600 nm measurements. Moreover, the feature maps for the principal components (PCs) are shown in Figure 9a,b.

The feature maps show the correlation between a PC and a specific feature, with darker colors signifying an inverse correlation and lighter colors signifying a direct correlation. That samples A and B are the ineffective substances for the measurements is further confirmed by PCA (Figure S6a,b), as all such measurements are grouped together irrespective of the interrogated bacteria. Moreover, it appears that the first PC can be employed to differentiate between the effects of the measured substances on the bacteria, with the results grouping together based on the interaction between the substances and the bacteria. From both Figure 9a,b, it appears that the first PC is strongly correlated with the dilution measurements and negative blank, weakly correlated with the positive control and weakly inversely correlated with the blank measurements. However, this is similar to the formula (Equation (1)) employed for the efficiency calculations, with single wavelength measurements being directly correlated with the efficiency measure and multi-wavelength measurements being inversely correlated with it. Thus, *P.a* appears to be the most efficient followed by *E.c* and then *S.a*. Other principal components do not appear to have a large effect on the behavior of the substances. However, this is to be expected, as the dataset appears to have little explained variance in those other metrics.

#### 2.5.2. Hydrogels with SAg/NPs-WT

The physicochemical properties, the good antimicrobial effect and the ease of formulation in such combinations informed the decision of using only SAg/NPs-WT in hydrogels and ointments for tests of antimicrobial activity.

**Figure 10:**
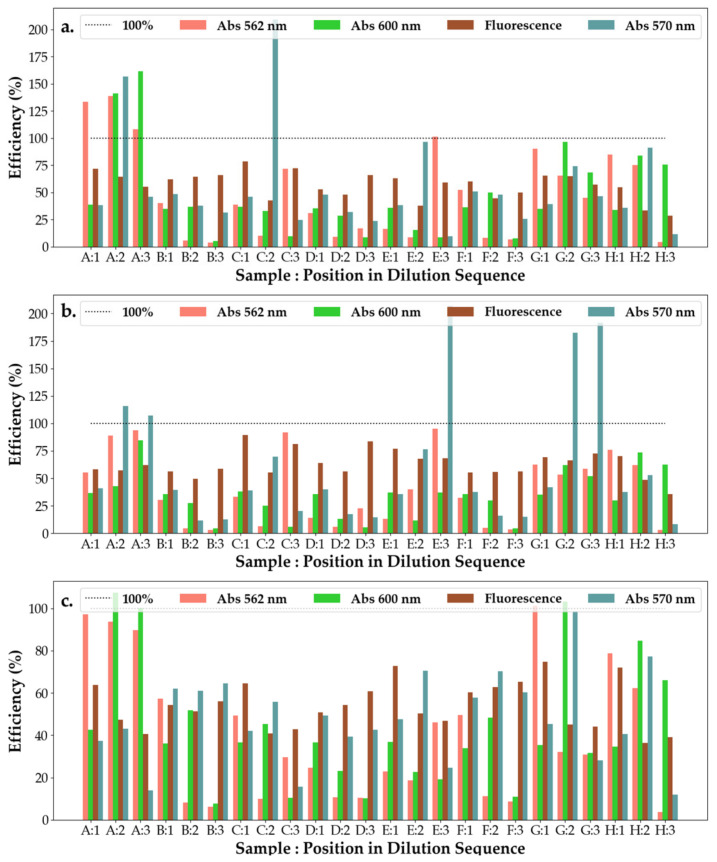
Efficiency of the antibacterial response (η) of the SAgNP-WT hydrogel samples at three levels of dilution tested against (**a**) *Escherichia coli*, (**b**) *Pseudomonas aeruginosa* and (**c**) *Staphylococcus aureus*. Samples A–H represent the following solutions tested against the bacteria: A—hydrogel control; B—AgNO_3_ + PVP + PEG; C—hydrogel A, D—AgNO_3_ + PVP + Gly; E—hydrogel B; F—AgNO_3_ + PEG + Gly; G—hydrogel C; and H—AgNO_3_ 1N, as also shown in Figure S5.

From Figure 10, it can be observed that all hydrogels have low efficiency on *S.a*, with the efficiency of the solutions decreasing across all measured wavelengths. For most responses (except C, D and E) of the hydrogel solutions to *E.c*, their efficiency, when investigated under 562 nm, is much higher than for their efficiency investigated under 600 nm. This suggests that the mechanics behind the efficiency change with the increase in dilution, whereas for the three exceptions the opposite is true, with the primary mechanic of reaction (which responds to 600 nm) becoming almost unresponsive upon further dilutions. At the second dilution, however, the main mechanism of efficiency is given by that responding most strongly to 570 nm illumination. For *P.a*, it appears that the mechanism responding to 570 nm is the main avenue of efficiency for hydrogel substances E and G, whereas for all but H, the main mechanism of efficiency is that which corresponds to 562 nm. For H, its efficiency under 600 nm increases with the repeated dilutions.

It can be seen from Figure S7a,b that solution H is the most responsive to a change in dilution, with it having the largest variation from the mean, as shown by the first principal component. However, this response for H seems to come irrespective of the interaction with a specific cell culture. Moreover, PC2 serves as a classifier for the response of the hydrogel solution to *S.a*, since on the third dilution, no hydrogel solutions have a significant efficiency when measured under 562 nm and 600 nm. PC3 also seems to differentiate between the effects of the hydrogel on P.a, with responses being closer to the blank and negative blank both under 562 nm light and 600 nm excitation for this interaction. From Figure S8a,b, it can be seen again that H does not seem to be varying with the cell culture; hence, it could be employed as a reference measurement to all other substances. PC2 correlates to a strong response to 570 nm light and, thus, can be employed to differentiate the response to *P.a* from all other cell cultures.

#### 2.5.3. Ointments with SAg/NPs-WT

**Figure 11:**
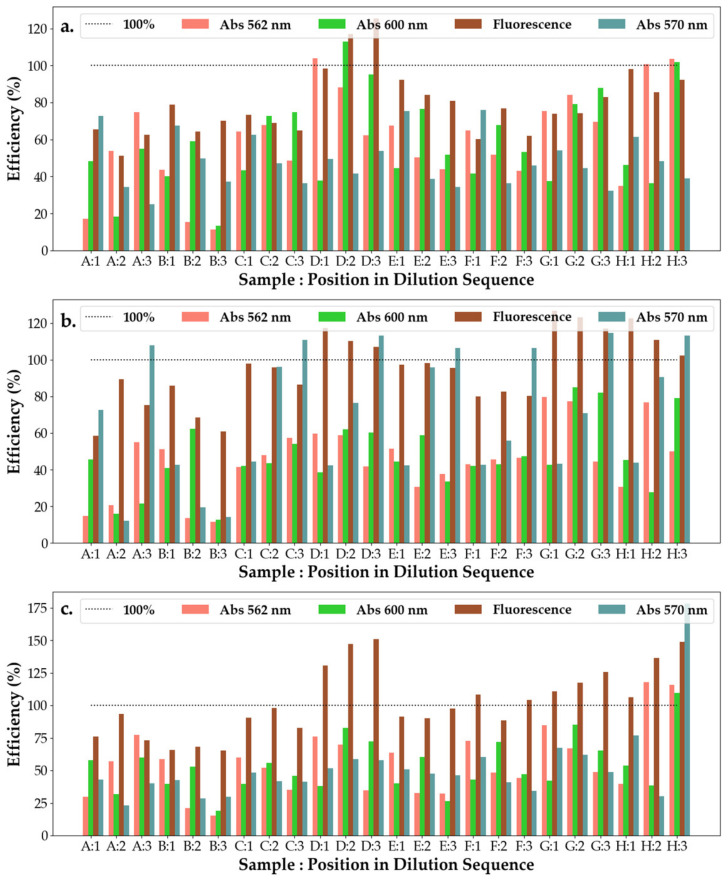
Efficiency of the antibacterial response (η) of the SAgNPs-WT ointment samples at three levels of dilution tested against (**a**) *Escherichia coli*, (**b**) *Pseudomonas aeruginosa* and (**c**) *Staphylococcus aureus*. Samples A–H represent the following solutions tested against the bacteria: A—Tween 20; B—Ont 1 + SAgNPs-WT 10S + 1% tween 20; C—Ont 1 + SAgNPs-WT 10S + 1% tween 20 (1:1:1); D—Ont 1 + SAgNPs-WT 10S + 1% tween 80 (1:1:1); E—Ont 2 + SAgNPs-WT 10S + 1% tween 80; F—Ont 2 + SAgNPs-WT 10S + 1% tween 20 (1:1:1); G—Ont 2 + SAgNPs-WT 10S + 1% tween 80 (1:1:1); and H—Tween 80, as also shown in Figure S5.

From Figure 11, it can be seen that the ointment efficiency measured using 570 nm excitation increases with the dilution of the substances strongly when interacting with *P.a*, whereas there was not a significant response to the interaction with the other two cell cultures. With the exception of A and H, the ointment solutions see a decrease in efficiency when measured with 562 nm light, while the efficiency measured with 600 nm light increases when interacting with the *S.a* cell culture. The opposite is true for solutions A and H. On interacting with *E.c*, the ointment solutions experience a decrease in efficiency with dilution, whereas all solutions experience variations in efficiency with dilutions without respecting a trend. Across all cell cultures, however, ointment D shows a high efficiency under all light measurements.

From the PCA measurement for the ointment presented in Figure S9a,b, there does not appear to be a cluster of all three cell cultures that is sufficiently close to suggest a reference solution. However, under 562 nm and 600 nm excitation, no ointments appear to be highly selective. PC2, which differentiates between the third and first dilutions, appears to serve as a good indicator of the efficiency of the ointments against *P.a*, with the only outlier being ointment B. This corresponds to the data observed in the bar plots when only solution B has an overall decrease in efficiency for the 562 nm and 600 nm excitation. Moreover, PC3 presents a better split for the identification of the response to *P.a*, with an equal response to both 562 and 600 nm illumination in the first dilution, setting the response to P.a apart from that of the other cell cultures. For 570 nm and fluorescent measurements (Figure S10a,b), PC1 shows a good split between the response to *P.a* and the other cell cultures due to the strong increase in efficiency with dilution when exposed to 570 nm excitation. The only exception is ointment B, which experiences a decrease in efficiency under 570 nm illumination with dilution. Similarly, PC2, which shows the comparison between fluorescence and 570 nm illumination, serves as a good classifier for the response to *S.a*, with it being characterized by consistently high differences between the two measurements across all dilutions.

### 2.6. Biological Tests Results

**Figure 12:**
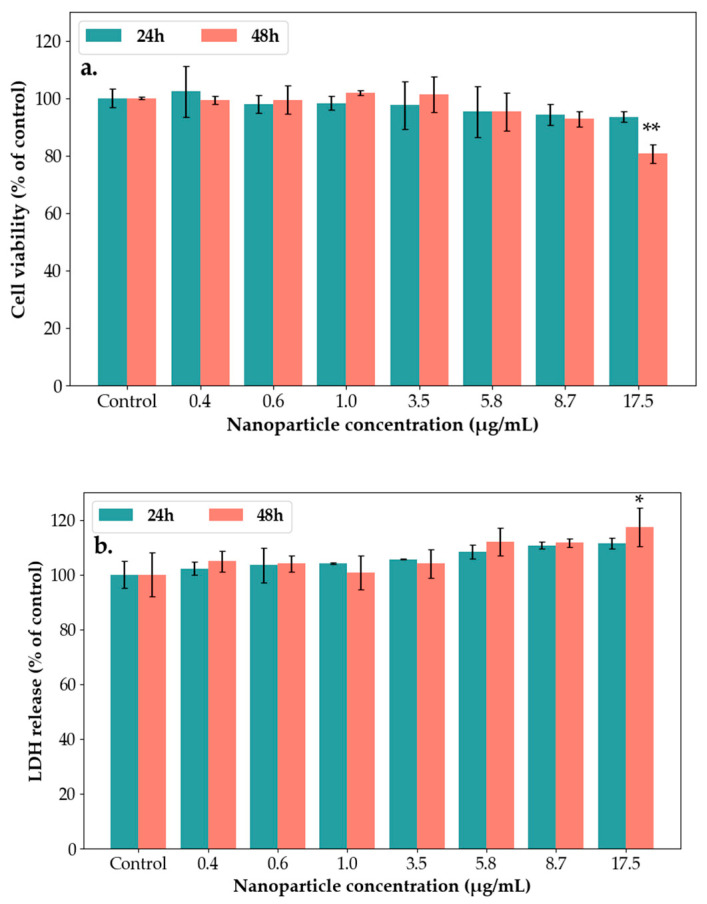

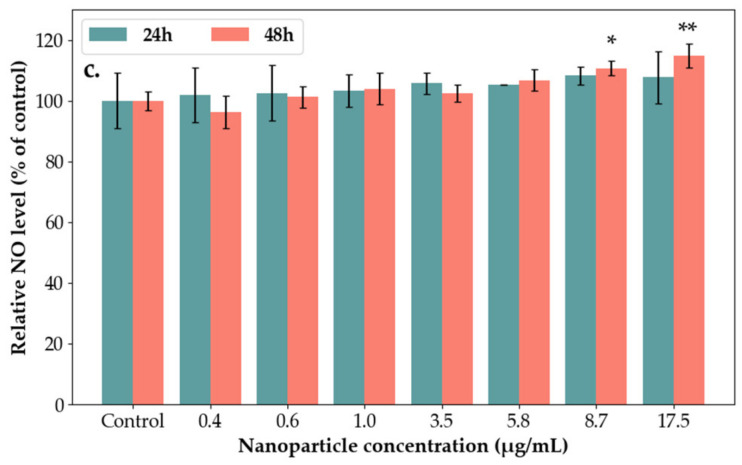
Biological tests performed on SAg/AuNPs-WT using the following techniques: (**a**) MTT assay after 24 h and 48 h of exposure to different concentrations of SAgNPs-WT in keratinocytes. Viability percentage was normalized to the untreated cells (without SAgNPs-WT). Results are calculated as mean ± SD (n = 3) and expressed as a percentage (%) of the control. (**b**) The lactate dehydrogenase released after 24 h and 48 h of exposure to different concentrations of SAgNPs-WT in HaCaT cells. Results are calculated as mean ± SD (n = 3) and expressed as a percentage (%) of control. (**c**) Effects of SAgNPs-WT on production of nitric oxide (NO) in HaCaT cells. Cells were treated with SAgNPs-WT (0.4–17.5 µg/mL) for 24 h and 48 h. Results are calculated as mean ± SD (n = 3) and expressed as a percentage (%) of the control. ** *p* < 0.01, * *p* < 0.05.

The cytotoxicity of SAgNPs-WT was evaluated by MTT and LDH assays using the HaCaT cell lines (Figure 12a,b). The inflammatory response was analyzed using the Griess method [48]. At 24 and 48 h, after the exposure of the keratinocytes to different concentrations of NPs (0.4–17.5 µg/mL), the production of nitric oxide from the culture medium was tested, and it is presented in Figure 12c.

In Figure 12a, the MTT results showed about a 20% decrease after 48 h at 17.5 µg/mL concentrations of NPs compared to the control level. No significant changes were registered for the other concentrations.

The level of LDH released in the culture media was increased after 24 and 48 h at the highest concentrations (Figure 12b). As shown in Figure 12c, the exposure of HaCaT cells to 8.7 µg/mL and 17.5 µg/mL concentrations of NPs induced a significant increase in NO compared to the control after 48 h. These results are in accordance with two other cytotoxicity studies regarding exposure of silica nanoparticles to normal human dermal fibroblasts (NHDF) and normal human epidermal keratinocytes (NHEK) [30]. These studies showed that silver nanoparticles are non-toxic and had no significant effect on cell survival profiles up to 24 h.

## 3. Materials and Methods

### 3.1. Plant Material and Preparation of the Plant Extract

*Salix alba L.* bark was collected from Dâmboviţa County, Romania, in March–April 2019. A backup specimen of the plant species was planted at the “Dimitrie Brândză” Botanical Garden, Bucharest, under the 409156 number tag (Figure 1a).

The willow bark was thoroughly washed with deionized water and allowed to fully dry at room temperature. After drying, it was weighted with a reading of 5 g obtained. The material was then placed in a Berzelius beaker, and 100 mL of deionized water was poured over it. The mixture was then placed in a stirrer plate and heated for 5 min using the thermometer to ensure that the temperature did not exceed 80 °C. After heating, the beaker was covered with a watch bottle and left to rest for 15 min. A cellulose filter and a conical funnel were then used to separate solid plant material from the aqueous extract.

### 3.2. Biosynthesis of SAg/AuNPs-WT

Two 80 mL solutions: one of 0.5 mM silver nitrate and one of 0.1 mM gold (III) chloride trihydrate (HAuCl_4_ × 3 H_2_O) (purchased from Sigma-Aldrich, Bucharest, Romania) were placed in Berzelius beakers. The aqueous extracts obtained in the previous step were then poured into 25 mL burettes with closed stopcocks, which were then placed on top of the beakers with the silver nitrate solution and the gold chloride solution. Each Berzelius beaker was then placed on the magnetic stirrer and heated. When the temperature of the two solutions reached 60 °C, the willow extract from the burette started dripping at a rate of 4–5 drops per 30 seconds. During synthesis, 11 vials were prepared and numbered for taking samples. At each color change, approximately 2 mL of the solution was sampled into one of the vials. A volume of 25 mL of extract was added to the 80 mL of 0.5 mM silver nitrate and 0.1 mM gold (III) chloride trihydrate, respectively. Each vial was then analyzed using the UV–Vis spectrophotometer, with the remainders of the silver and gold solutions used for physicochemical characterization and to testi their biological (antimicrobial and anti-psoriasis effect) activity.

### 3.3. Preparation of Formulations

- Ointment with SAgNPs-WT

Solid components (lanolin and Vaseline) were weighed and melted using a water bath (at 60–70 °C) and mixed to homogenization. The nanoparticle solution was heated to 50 °C and was gradually introduced into the fluidized and homogenized lipophilic phase and mixed until fully cooled. This resulted in a semi-solid pharmaceutical preparation for topical application to the skin for protection. The result’s appearance is that of a homogeneous, yellowish-white ointment with a characteristic odor. The product was stored in tightly closed containers at temperatures of up to 25 °C. It was subsequently tested in hydrogels with SAgNPs-WT

Two solutions—one (S1) formed by dissolving 1 g of chitosan (CS) in 100 mL of 2% aqueous acetic acid with an added 0.3 mL of glycerol and another (S2) formed by adding of 2.5 g of gelatin in 100 mL of deionized water and dissolved at 40 °C with an added 0.7 mL of glycerin—were prepared. Solution S1 was mixed with solution S2 at different volumetric ratios (S1:S2), resulting in three separate mixtures (A—5:5; B—3:7; C—1:9), with 5 mL of silver nanoparticles biosynthesized from *Salix alba* and 0.3 mL of 0.25% Glutaraldehyde (GA) added in each of the mixtures. This procedure resulted in odorless, reddish-brown and homogeneous hydrogels, as seen in Figure S11 (Supplementary Materials). The hydrogels were stored in Petri dishes at temperatures of up to 50 °C until testing. All three samples were used for antimicrobial and physicochemical testing.

### 3.4. Antimicrobial Activity

- Diffusimetric antibiogram

Testing of the substances was carried out by a qualitative method (screening) adapted from the diffusimetric antibiogram method—the modified Kirby–Bauer technique—by seeding the surface of the Mueller–Hinton solid medium with the bacterium of interest and applying the test substance to measure the diameter of the zone of inhibition (ZOI) in the SIR system (sensitive, intermediate, resistant). The working technique consisted of putting these substances in contact with the pathogenic bacteria. Thus, negative controls (sterile non-impregnated Ø 6 mm standard paper disc) and samples consisting of sterile standard paper discs with Ø 6 mm impregnated with 10 µL of test solution, sterile standard paper discs with Ø 9 mm impregnated with 20 µL of test solution and a borosilicate glass cylinder loaded with 100 µL of test solution were employed. The plates with the test samples were incubated at 37 °C and were read at 24 and 48 h after preparation by calculating the average diameters of the bacterial inhibition zones and expressed in tenths of a millimeter.

- Plate microdilution

The microbiological testing of pharmaceutical substances with metal nanoparticles obtained by “green synthesis” was performed comparatively on microplates with 96 wells (96-well CytoOne^®^ plate), which were incubated aerobically at 37 °C for 24 h according to the “Gold Standard” for determining the antibacterial and antibiofilm effect of the tested substances [49]. Absorbances at different wavelengths were measured spectrophotometrically with *EnSight Multimode Plate Reader* computerized equipment PerkinElmer, (Waltham, Massachusetts, United States). Measurements were made for the antimicrobial effect at 562 nm and 600 nm, and for the antibiofilm effect, after coloration of the biofilm with crystal violet 1%, at 570 nm for absorbance and at 550 nm (excitation) with 640 nm (emission) for fluorescence. The device software automatically calculated the arithmetic mean of the sample for each well. Due to studying biological samples, where individual variability and dynamics over time are much higher than in chemical samples, we compared arithmetic means for 3 tests with bacterial culture separately, but on the same plate, to avoid technical errors. In the case of hydrogels with silver nanoparticles, stability testing was also performed to compare the percentage effectiveness after one year of using the same measurement protocol. 

Each row of microplates (A–H) was reserved for a substance as follows: at no. 1, blank (physiological serum 200 µL); at no. 2, the negative control (physiological serum 50 µL, substance dilution 50 µL and Mueller–Hinton broth 100 µL); at no. 3, the positive control (physiological serum 50 µL, bacterial suspension 50 µL and Mueller–Hinton culture medium 100 µL); at no. 4, 5 and 6, the sample at the first dilution (first decimal dilution 50 µL, bacterial suspension 50 µL and Mueller–Hinton culture medium 100 µL); at no. 7, 8 and 9, the sample at the second dilution (second decimal dilution 50 µL, bacterial suspension 50 µL and Mueller–Hinton broth 100 µL); at no. 10, 11 and 12, the sample at the third decimal dilution (third dilution 50 µL, bacterial suspension 50 µL and Mueller–Hinton culture medium 100 µL). In this way, an active substance concentration (mother solution) of 25% at D0, 2.5% at D1, 0.25% at D2, 0.025% at D3 was achieved to establish a concentration/effect correlation (Q/E). The most sensitive result for the antimicrobial effect was at 600 nm, where the highest overall arithmetic mean of absorbance was recorded. Antibacterial efficacy on mathematical intervals was quantified as follows: very good: >75%; good: 50–75%; satisfactory: 25–49%; unsatisfactory: 0–24%; and percentage efficacy with negative values was interpreted as favoring bacterial growth and/or bacterial biofilm.

#### The Bacterial Strains

The selected and semi-preserved bacterial strains introduced in the study came from the sub-collection of the Experimental Microbiology Laboratory of the “Cantacuzino” National Military Medical Institute for Research and Development, Bucharest, Romania, as a result of the laboratory’s research activity on multi-resistant strains from clinical samples. Representative species were chosen for the main groups of pathogenic bacteria (the most common in human and veterinary infectious pathology, in hospital flora, in nosocomial infections and in multidrug-resistant infections): *Staphylococcus aureus* (*S.a*), for gram-positive cocci; *Escherichia coli* (*E.c*), for gram-negative bacilli, enterobacteria; *Pseudomonas aeruginosa* (*P.a*), for gram-negative bacilli, non-enterobacteria.

### 3.5. The Samples

The following substances were tested for the in vitro evaluation of the antimicrobial activity of pharmaceutical substances with metal nanoparticles obtained by “green synthesis”: positive controls—AgNO_3_ 1N; negative controls—glycerin anh. (Belgia), glycerin anh. P.a (Sanimed); blank controls—physiological serum; samples collected during the “green synthesis” of the nanoparticles solutions obtained from willow bark:

WT based on chitosan and glycerin: hydrogel A, B and C (see hydrogels SAgNPs-WT preparation); ointments with SAgNPs-WT: ointment + 1% Tween 20; and ointment with SAgNPs-WT + 1%Tween 80. Figure S4 shows a diagram of the plates and the samples for each well.

### 3.6. Evaluation of the Antimicrobial Efficiency

Antimicrobial efficiency (η) of the substance of interest was analyzed using Equation (1):

$$\eta =\left(1-\frac{{8\;\times \mu }_{\mathrm{Dilution}}}{\sum_{A}^{H}{\mathrm{Blank}}_{-}+\sum_{A}^{H}{\mathrm{Blank}}_{+}-\;\sum_{A}^{H}{\mathrm{Blank}}_{0}}\right)\times \;100$$

where $\sum_{A}^{H}{\mathrm{Blank}}_{-}$ represents the summation of the negative blank absorbances of the 8 different substances, $\sum_{A}^{H}{\mathrm{Blank}}_{+}$ represents the summation of the positive blank absorbances of the 8 different substances, $\sum_{A}^{H}{\mathrm{Blank}}_{0}$ represents the summation of the blank absorbances of the 8 different substances, and $\mu_{\mathrm{Dilution}}$ represents the mean absorbance measurement for the 3 repeats of each dilution measurement. The antimicrobial efficiency can also be expressed as (1 *−* Bacterial Survivability) × 100.

Principal component analysis (PCA) was also conducted in Python 3.6 using the *sklearn* library. This technique involves summarizing the target dataset in a n-dimensional space, where each component is perpendicular to the others, such that the variance in the data projections on each component can be analyzed. Analysis was performed separately for the multi-wavelength and single wavelength excitation cases. A feature vector for the analysis of the absorbance data consisted of the blank measurements and the three repeats of each of the three dilution stages for both channels of the measurements: 562 nm and 600 nm for the single wavelength excitation and fluorescence and absorbance (centered at 580 nm) for the multi-wavelength excitation. Thus, a 1 × 24 vector was generated for each solution, and given that 8 substances were tested for each of the three bacteria, this led to a 24 × 24 feature space.

### 3.7. Characterizations Methods

Absorption properties of SAgNPs-WT during “green synthesis” were checked using a U-0080D UV–Vis spectrometer Hitachi (Tokyo, Japan), while fluorescence emission spectra were recorded with a spectrometer equipped with a 450 W Xenon lamp as an excitation source (model FLS920, Edinburgh Instruments Ltd., UK).

Nanoparticles were investigated morphologically using Nova NanoSEM 630—a field emission scanning microscope (FE-SEM) FEI Company (Hillsboro, OR, USA) with an operating voltage of 5 kV and a magnitude of 40 and 300 kx.

The structural features (crystalline structure, crystallite size) were investigated using a 9 kW rotating anode X-ray diffraction system (SmartLab, Rigaku, Japan) that uses Cu Kα1 radiation (λ = 1.54056 Å). Analysis was performed using a grazing incidence X-ray diffraction (GIXRD) technique at small angles of incidence (0.5°).

Bruker Optics Tensor 27 spectrometer was used to study the chemical bond configuration in nanomaterial samples by Fourier transform infrared (FT-IR) spectrometry. Spectra were plotted at room temperature in the 4000–400 ${\mathrm{cm}}^{-1}$ wavenumber range by averaging 64 scans, with a resolution of 4 ${\mathrm{cm}}^{-1}$ attained, using an ATR (attenuated total reflection) Platinum holder. Aqueous samples were dehydrated to remove water molecules that could disrupt the spectrum.

### 3.8. Biological Tests

The silver nanoparticles (Ag-NPs) were tested on human keratinocyte cell lines (HaCaT). The cells were grown in DMEM (Dulbeco’s Modified Eagle Medium) culture medium supplemented with 2 mM L-glutamine, 100 units per milliliter Penicillin/Streptomycin/Amphotericin and 10% fetal bovine serum, maintained in a humidified incubator at 37 °C, with a concentration of 5% CO_2_. HaCaT cells were seeded at a density of $3\times 10^{4}$ cells/mL in 98-well plates. After the adhesion of the cells, they were tested with different concentrations of SAgNPs-WT for 24 and 48 h. Untreated cells were used as a control. In this study, the MTT, LDH and NO tests were presented.

#### 3.8.1. MTT Assay

Cell viability was determined using the MTT (3-(4,5-dimethylthiazol-2-yl)-2,5-diphenyltetrazolium bromide) colorimetric assay. After treatment, cells with different concentrations (5, 10, 25, 50 and 100 µg/mL) of nanoparticles were incubated 24 and 48 h, respectively [50]. Then, the culture medium was removed, and each well was washed with tampon phosphate saline. After that, 150 µL of MTT (1 mg/ml) was added. After 2 h of incubation, MTT was removed, and the formazan crystals were solubilized in 100% isopropanol. The optical density was measured at 595 nm using a FlexStation 3 Multi-Mode Microplate Reader.

#### 3.8.2. LDH Release

An LDH (lactate dehydrogenase) test was conducted according to the In Vitro Toxicology Assay Kit, Lactic Dehydrogenase (Sigma-Aldrich). A volume of 50 µL of culture medium was homogenized with 100 µL of reaction mix (substrate, cofactor and dye in a 1:1:1 ratio). After 20 min at room temperature, the reaction was stopped with 1N HCl (1/10 of the volume), and the absorbance was read at 450 nm using a FlexStation 3 Multi-Mode Microplate Reader from Molecular Devices LLC and SoftMax Pro 7.1 software.

#### 3.8.3. NO Production

An amount of 80 µL of culture medium from each sample was treated with a mixture of sulfanilamide and naphthyl-ethylene-diamine solution in a 1:1 ratio. Optical density was read at 540 nm using the Flex Station 3 Multi-Mode Microplate Reader from Molecular Devices LLC and SoftMax Pro software. All results were expressed as mean ± SD (standard deviation) for the three independent experiments. Statistical analysis was performed by Student’s t-test. A p-value of less than 0.05 was considered statistically significant.

## 4. Conclusions

This article successfully presented the development and characterization of green nanoparticles extracted from the *Salix alba* tree, their antimicrobial efficiency in formulations and their potential as topical pharmaceutical substances for psoriasis wounds. It was observed that all metallic nanoparticles had antibacterial and antibiofilm efficacy on *S.a*, with very good antibiofilm efficacy also appearing in hydrogels with silver nanoparticles. The antibacterial and antibiofilm efficacy of some metal nanoparticles on *E.c* was similar to that obtained on gram-positive cocci, with antibiofilm efficacy also observed in the hydrogels that made use of SAgNPs-WT loaded with chitosan and gelatin. The best antibacterial and antibiofilm efficacy on *P.a*, a particularly aggressive bacterium that very often shows resistance to antibiotics, was obtained using the hydrogels with silver nanoparticles, with SAgNPs-WT having the best antibiofilm effectiveness (over 75%) on *P.a* and a 200% increased bacterial efficiency when compared to controls and their individual components. Although the gold nanoparticles showed important antimicrobial activity on all three bacteria, the identified physicochemical properties, such as an increased propensity to flocculate over time, made them unsuitable for formulations, such as hydrogels or ointments, as they require treatment or functionalization with chemical agents to be stabilized. Meanwhile, green-synthesized silver nanoparticles were a good candidate for such formulations due to their stability expressed under the physicochemical analysis performed in this study.

All nano formulations, with or in combination with silver nanoparticles, obtained by “green synthesis” were tested as potential antimicrobial or keratinocyte-acting substances. For the concentrations evaluated for antimicrobial activity, the cells in contact with the SAgNPs-WT did not present any signs of cytotoxicity. Compounds containing green-synthesized silver nanoparticles were, thus, shown to be effective antimicrobial agents, with anti-infective drugs, hygienic and cosmetic products based on them leading to potential improvements in the treatment of psoriasis wounds. This could lead to visible improvements in the lives of the highly stigmatized patients suffering from psoriasis.

## Figures and Tables

**Table 1:** SIR interpretation of the mean diameters of the inhibition zone in the diffusimetric antibiogram for metal nanoparticles tested on bacteria.

No.	Samples	Bacteria Strains									SIR *
		*Staphylococcus aureus*			*Escherichia coli*			*Pseudomonas aeruginosa*			
		D0	D1	D2	D0	D1	D2	D0	D1	D2	
1.	SNPAu-WT 10S	0	0	0	0	0	0	0	7	7	Weak on P.a
2.	Ointment 1 with SNPAg-WT 10S + Tween 20, 1%	14.5	0	0	0	0	0	10.5	7	0	Good on S.a and weak on P.a
3.	Hydrogel A	0	0	0	0	0	0	8	7	0	Weak on P.a

Legend: *Staphylococcus aureus (S.a); Escherichia coli (E.c)*; and *Pseudomonas aeruginosa (P.a)*. * SIR interpretation: 0 = negative (no antibacterial effect) = resistant; <10 mm = weak antibacterial effect = intermediate; 10–15 mm = good antibacterial effect = sensitive; and >15 mm = very good antibacterial effect = very sensitive.

**Table 2:** Testing of substances by the adapted diffusimetric antibiogram method.

No.	Substance	Antibiogram Diffusimetric (mm)						
		*Staphylococcus aureus*		*Escherichia coli*		*Pseudomonas aeruginosa*		SIR Interpretation
		10 µL	100 µL	10 µL	100 µL	10 µL	100 µL	
1.	CS 1% in acetic acid 2% (D0)	7	19.5	0	22.5	10	15	Very Sensitive
2.	G 2.5% in water (D0)	0	0	0	0	0	0	Does not diffuse
3.	CS + G (D1)	0	0	10 (MR)	20 (MR)	0	0	Does not diffuse
4.	Hydrogel: A (D1)	0	0	0	0	0	22.5	Does not diffuse
5.	SAgNPs-WT 10S	0 *	8	0	17.5	20	24	* Colors the culture medium orange Very Sensitive

* SIR interpretation: 0 = negative (no antibacterial effect) = resistant; <10 mm = weak antibacterial effect = inter-mediate; 10–15 mm = good antibacterial effect = sensitive; and >15 mm = very good antibacterial effect = very sensitive.

## Data Availability

Data are contained within the article and supplementary materials.

## Supplementary Materials

The following supporting information can be downloaded at: https://www.mdpi.com/article/10.3390/ph16010048/s1, Figure S1a: SAgNPs-WT distribution with N = 389; Figure S1b: SAuNPs-WT distribution with N = 375; Figure S2: UV–Vis spectra of 1. Initial colloidal solution with SAgNPs-WT, 2. After one year of storage at room temperature protected from light; Figure S3: UV–Vis spectra showing the effect of varying pH on stability of SAgNPs-WT. The inset photo shows the change in color with different pH corresponding to absorption spectra of the gold nanoparticles; Figure S4: SEM image of the hydrogel based on CS/gelatin and SAgNPs-WT; Figure S5: The scheme of the plate and the tested samples, each on the one plate used for antimicrobial tests; Figure S6a: PCA representation for the single wavelength measurements for SAg/AuNPs-WT; Figure S6b: PCA representation of the multi-wavelength measurements for SAg/AuNPs-WT; Figure S7a: PCA representation for the single wavelength measurements for hydrogels; Figure S7b: PCA representation of the multi-wavelength measurements for hydrogels; Figure S8a: Feature map for the single wavelength measurements for hydrogels; Figure S8b: Feature map for the multi-wavelength measurements for hydrogels; Figure S9a: PCA representation for the single wavelength measurements for ointments; Figure S9b: PCA representation of the multi-wavelength measurements for ointments; Figure S10a: Feature map for the single wavelength measurements for ointments; Figure S10b: Feature map for the multi-wavelength measurements for ointments; Figure S11: Hydrogels with: A. chitosan-gelatin-SAgNPs volume ratio = 3:7 and 5 mL SAgNPs, B. ratio = 5:5 (*v/v*), C. ratio = 1:9; Table S1: FT-IR possible assignments of the colloidal solution (dehydrated) of SAgNPs-WT; Table S2: FT-IR possible assignments of the hydrogels based on CS/G and SAgNPs-WT; Table S3: SIR interpretation of mean inhibition zone diameters in diffusimetric antibiogram for chemicals tested on bacteria.
