# Supplementary material for: The Physicochemical and Antimicrobial Properties of Silver/Gold Nanoparticles Obtained by “Green Synthesis” from Willow Bark and Their Formulations as Potential Innovative Pharmaceutical Substances
Source: Pharmaceuticals (Basel). 2022 Dec 29;16(1):48. doi: 10.3390/ph16010048 (PMC9867178; doi:10.3390/ph16010048)
Supplement: Supplementary file 1 [file pharmaceuticals-16-00048-s001.zip › pharmaceuticals-2079855-supplementary.pdf]

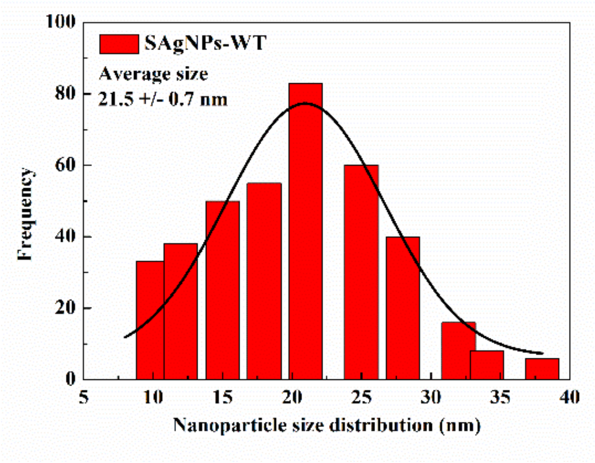

Figure S1a. SAgNPs-WT distribution with N=389

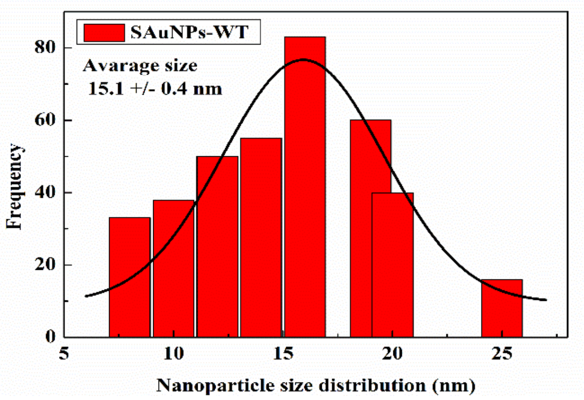

Figure S1b. SAuNPs-WT distribution with N=375

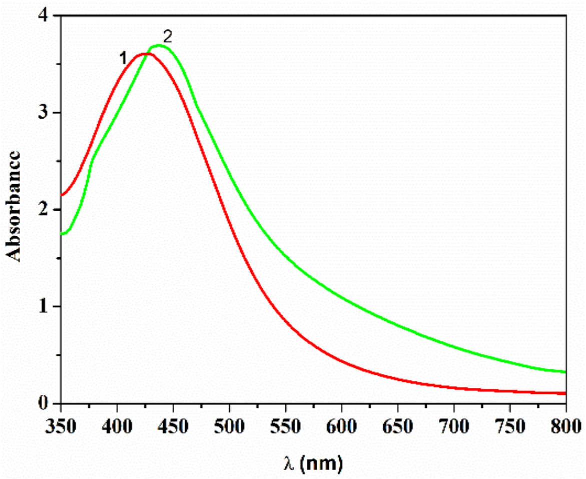

Figure S2. UV-VIS spectra of: 1. Initial coloidal solution with SAgNPs-WT; 2. after one year of store at room temperature protected from light

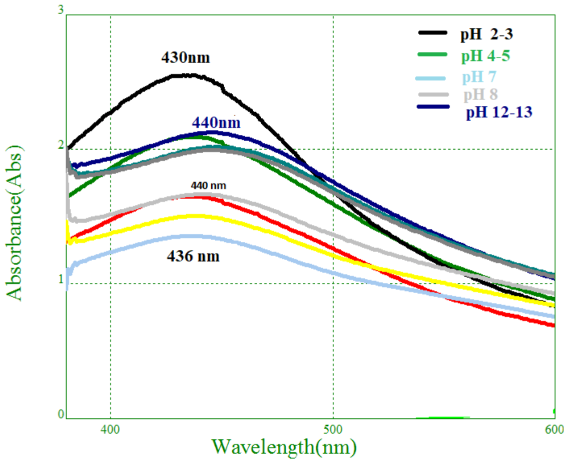

Figure S3. UV–Vis spectra showing the effect of varying pH on stability of SAgNPs-WT. The inset photo shows the change in color with different pH correspond to absorption spectra of the gold nanoparticles

Table S1. FT-IR possible assignments of the colloidal solution (dehydrated) of SAgNPs-WT

Possible assignments	Possible chemical compounds	Wavenumber (cm^{-1})			
		Willow extract	SAgNPs-WT (after 1 day)	SAgNPs-WT (after 7 days)	SAgNPs-WT (after 180 days)
**O-H, ν**	Alcohols and phenols hydroxyl groups	3306	3259	3275	3271
**=C-H, ν**	Aromatic ring	3022	3012	3013	3013
**-C-H, ν**	Aliphatic groups	2980	2976	2978	2978
**-C-H, ν**	Aliphatic groups	2915	2913	2919	2917
**C=O, ν**	Carbonyl groups	1725	1710	-	-
**C=O+ C=C, ν**	C=O conjugated to the aromatic ring	1602	1604	1626	1616
**C=C, ν**	Aromatic skeletal	1516	1519	1523	1521
**-C-H, δ**	Aliphatic groups	1444	-	-	-
**C-O, ν**	Phenols compounds	1395	1397	1399	1397
**C-O, ν**	Guaiacol and syringyl rings	1240	1261	1262	1260
**C-O, δ**	Alcohols or aliphatic ethers	1036	1024	1023	1020
**C-H, δ**	Aromatic ring	818	818	802	819
**C-H, δ**	Aromatic ring	773	-	-	-
**C-H, δ**	Aliphatic groups	736	-	-	-
**C-H, δ**	Aliphatic groups	610	-	-	-
**O-H, δ**	Alcohols and phenols hydroxyl groups	514	-	-	-
Note: ν - stretching and δ – bending vibrations					

Table S2. FT-IR possible assignments of the hydrogels based by CS/G and SAgNPs-WT

Possible bond assignments	From…	Wavenumber (cm^{-1})		
		Gelatina	Chitosan	Ag-Hydrogel
**N-H+O-H, ν+ν**	Amida A groups	3273	3269	3297
**=C-H, ν**	Alkenyl groups	3078	-	3077
**C-H, ν**	Amida B groups	2938	2935	2919
**C-H, ν**	Amida B groups	2878	2876	2873
**C=O, ν**	Amida I, N -acetyl groups	1634	1638	1636
**CN+NH, ν+δ**	Amida II, N -acetyl groups	1549	1553	1543
**CH+C-C, δ**	Skeletal	1454	-	1442
**C-O, ν**	Carboxyl groups	1404	1408	1398

**C-H, δ**	Aliphatic groups	-	1378	-
**C-N+C-H**	N-acetil groups	1336	1321	1336
**CN+NH, ν+δ**	Amida III groups	1280	-	-
**CN+NH, ν+δ**	Amida III groups	1240	1255	1237
**C-O-C, ν**	Carboxyl groups	1203	-	1197
**C-O–C, ν**	Carboxyl/ethoxy groups	1109	1152	1156
**C-O/C-O+C-C, ν**	Carboxyl/ethoxy groups	1037	1027	1030
**C-H, δ**	Aliphatic groups	922	-	-
**C-H, δ**	Aliphatic groups	849	851	-
**NH+C–O, δ**	N -acetyl groups	-	557	-

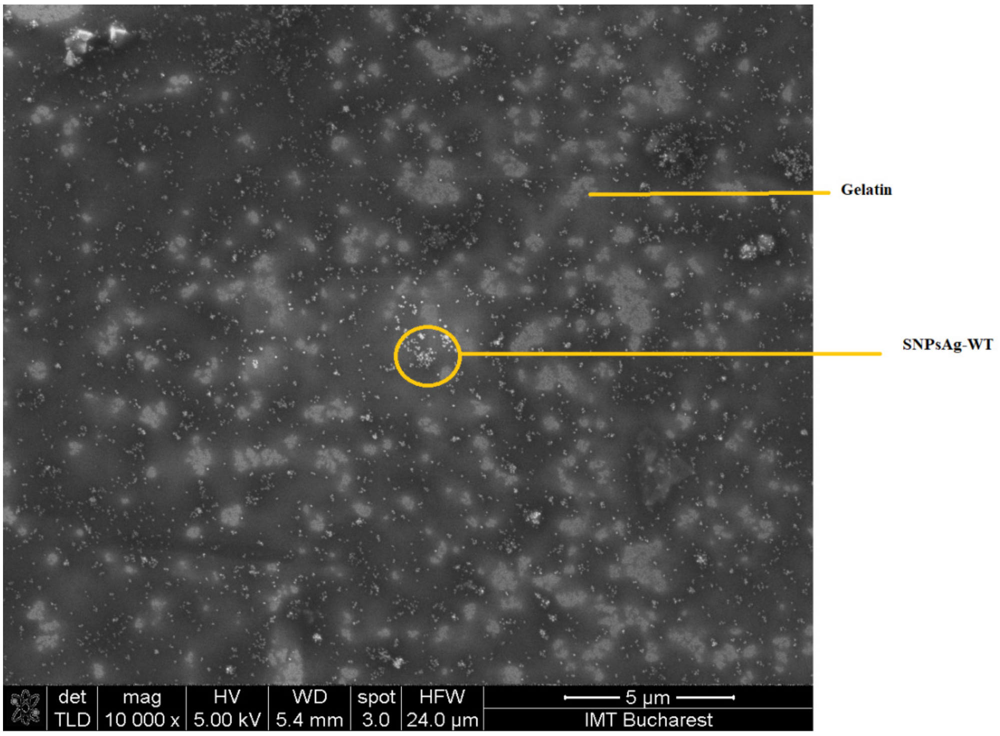

Figure S4. SEM image of the Hydrogel based by CS/G and SAgNPs-WT

	1	2	3	4	5	6	7	8	9	10	11	12	SAMPLES		
													Nanoparticles	Hydrogels	Ointments (Ont)
A	PS 200 µl	MHB: 100 µl PS: 50 µl S1-D1: 50 µl	MHB: 100 µl PS: 50 µl Bacteria: 50 µl	MHB: 100 µl Bacteria: 50 µl S1-D1: 50 µl	Idem 4	Idem 4	MHB: 100 µl Bacteria: 50 µl S1-D2: 50 µl	Idem 7	Idem 7	MHB: 100 µl Bacteria: 50 µl S1-D3: 50 µl	Idem 10	Idem 10	SAuNPs-WT 1S	Hydrogels Control	Tween 20
B	PS 200 µl	MHB: 100 µl PS: 50 µl S2-D1: 50 µl	MHB: 100 µl PS: 50 µl Bacteria: 50 µl	MHB: 100 µl Bacteria: 50 µl S2-D1: 50 µl	Idem 4	Idem 4	MHB: 100 µl Bacteria: 50 µl S2-D2: 50 µl	Idem 7	Idem 7	MHB: 100 µl Bacteria: 50 µl S2-D3: 50 µl	Idem 10	Idem 10	SAgNPs-WT 1S	AgNO3+PVP+PEG	Ont 1+SAgNPs-WT 10S+1% tween 20
C	PS 200 µl	MHB: 100 µl PS: 50 µl S3-D1: 50 µl	MHB: 100 µl PS: 50 µl Bacteria: 50 µl	MHB: 100 µl Bacteria: 50 µl S3-D1: 50 µl	Idem 4	Idem 4	MHB: 100 µl Bacteria: 50 µl S3-D2: 50 µl	Idem 7	Idem 7	MHB: 100 µl Bacteria: 50 µl S3-D3: 50 µl	Idem 10	Idem 10	Salix alba Extract	Hydrogel A	Ont 1+SAgNPs-WT 10S+1% tween 20 (1:1:1)
D	PS 200 µl	MHB: 100 µl PS: 50 µl S4-D1: 50 µl	MHB: 100 µl PS: 50 µl Bacteria: 50 µl	MHB: 100 µl Bacteria: 50 µl S4-D1: 50 µl	Idem 4	Idem 4	MHB: 100 µl Bacteria: 50 µl S4-D2: 50 µl	Idem 7	Idem 7	MHB: 100 µl Bacteria: 50 µl S4-D3: 50 µl	Idem 10	Idem 10	Salix alba Extract	AgNO3+PVP+Gly	Ont 1+SAgNPs-WT 10S+1% tween 80 (1:1:1)
E	PS 200 µl	MHB: 100 µl PS: 50 µl S5-D1: 50 µl	MHB: 100 µl PS: 50 µl Bacteria: 50 µl	MHB: 100 µl Bacteria: 50 µl S5-D1: 50 µl	Idem 4	Idem 4	MHB: 100 µl Bacteria: 50 µl S5-D2: 50 µl	Idem 7	Idem 7	MHB: 100 µl Bacteria: 50 µl S5-D3: 50 µl	Idem 10	Idem 10	SAuNPs-WT 7S	Hydrogel B	Ont 2+SAgNPs-WT 10S+1% tween 80
F	PS 200 µl	MHB: 100 µl PS: 50 µl S6-D1: 50 µl	MHB: 100 µl PS: 50 µl Bacteria: 50 µl	MHB: 100 µl Bacteria: 50 µl S6-D1: 50 µl	Idem 4	Idem 4	MHB: 100 µl Bacteria: 50 µl S6-D2: 50 µl	Idem 7	Idem 7	MHB: 100 µl Bacteria: 50 µl S6-D3: 50 µl	Idem 10	Idem 10	SAuNPs-WT 10S	AgNO3+PEG_Gly	Ont 2+SAgNPs-WT 10S+1% tween 20 (1:1:1)
G	PS 200 µl	MHB: 100 µl PS: 50 µl S7-D1: 50 µl	MHB: 100 µl PS: 50 µl Bacteria: 50 µl	MHB: 100 µl Bacteria: 50 µl S7-D1: 50 µl	Idem 4	Idem 4	MHB: 100 µl Bacteria: 50 µl S7-D2: 50 µl	Idem 7	Idem 7	MHB: 100 µl Bacteria: 50 µl S7-D3: 50 µl	Idem 10	Idem 10	SAgNPs-WT 7S	Hydrogel C	Ont 2+SAgNPs-WT 10S+1% tween 80 (1:1:1)
H	PS 200 µl	MHB: 100 µl PS: 50 µl S8-D1: 50 µl	MHB: 100 µl PS: 50 µl Bacteria: 50 µl	MHB: 100 µl Bacteria: 50 µl S8-D1: 50 µl	Idem 4	Idem 4	MHB: 100 µl Bacteria: 50 µl S8-D2: 50 µl	Idem 7	Idem 7	MHB: 100 µl Bacteria: 50 µl S8-D3: 50 µl	Idem 10	Idem 10	SAgNPs-WT 10S	AgNO3 1N	Tween 80

Legend: Physiological Serum (PS); Mueller Hinton Broth (MHB); Sample1, 2... (S1, 2...)

Blank | Positive Control | the sample at second dilution | Negative Control

Figure S5. The scheme of the plate and the tested samples each on the one plate use for antimicrobial tests

Table S3. SIR interpretation of mean inhibition zone diameters in diffusimetric antibiogram for chemicals tested on bacteria

No	Chemical substance tested	**Strains type**									**SIR**
		Stapylococcus aureus			***Escherichia coli***			***Pseudomonas aeruginosa***			
		**D0**	**D1**	**D2**	**D0**	**D1**	**D2**	**D0**	**D1**	**D2**	
**1.**	$AgNO_3$ 1N	24.5	16	13	20.5	12.5	12	27.5 (MR)	16	13.5	Staining of the culture medium at D0 and D1 Very sensitive on S.a., E.c P.a

Legend: *Stapylococcus aureus (S.a); Escherichia coli (E.c); Pseudomonas aeruginosa (P.a)*
SIR interpretation: 0 = negative (no antibacterial effect)= Resistant; <10 mm = weak bacterial effect = Intermediate; 10-15 mm = good antibacterial effect = Sensitive; >15 mm = very good antibacterial effect = Very sensitive

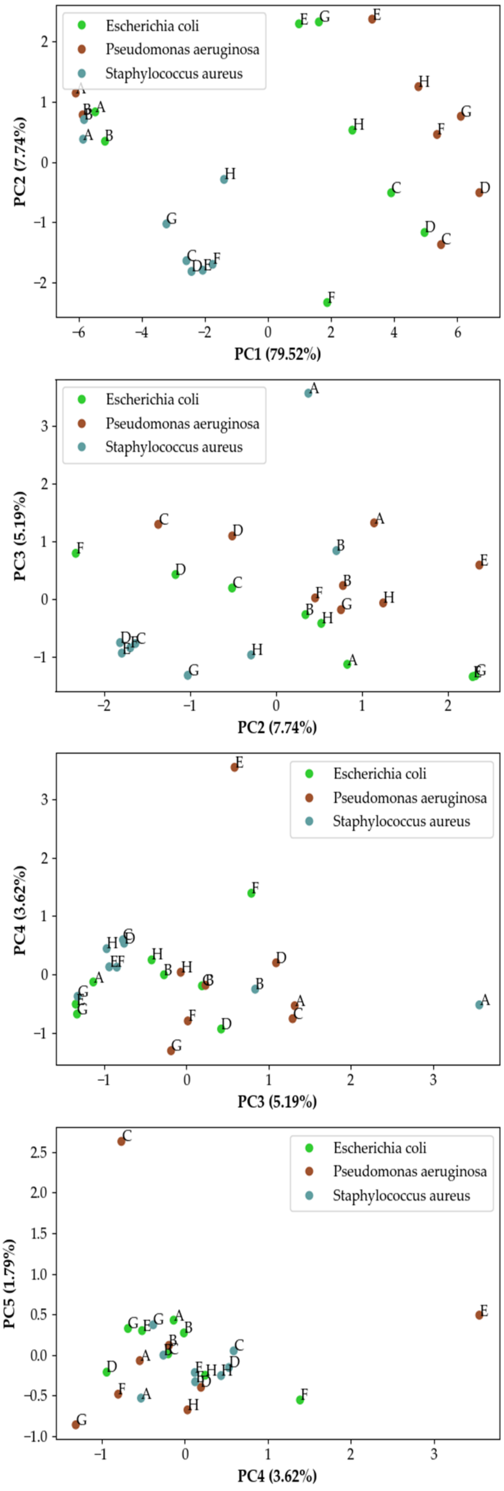

Figure S6a. PCA representation for the single wavelength measurements for SAg/AuNPs-WT

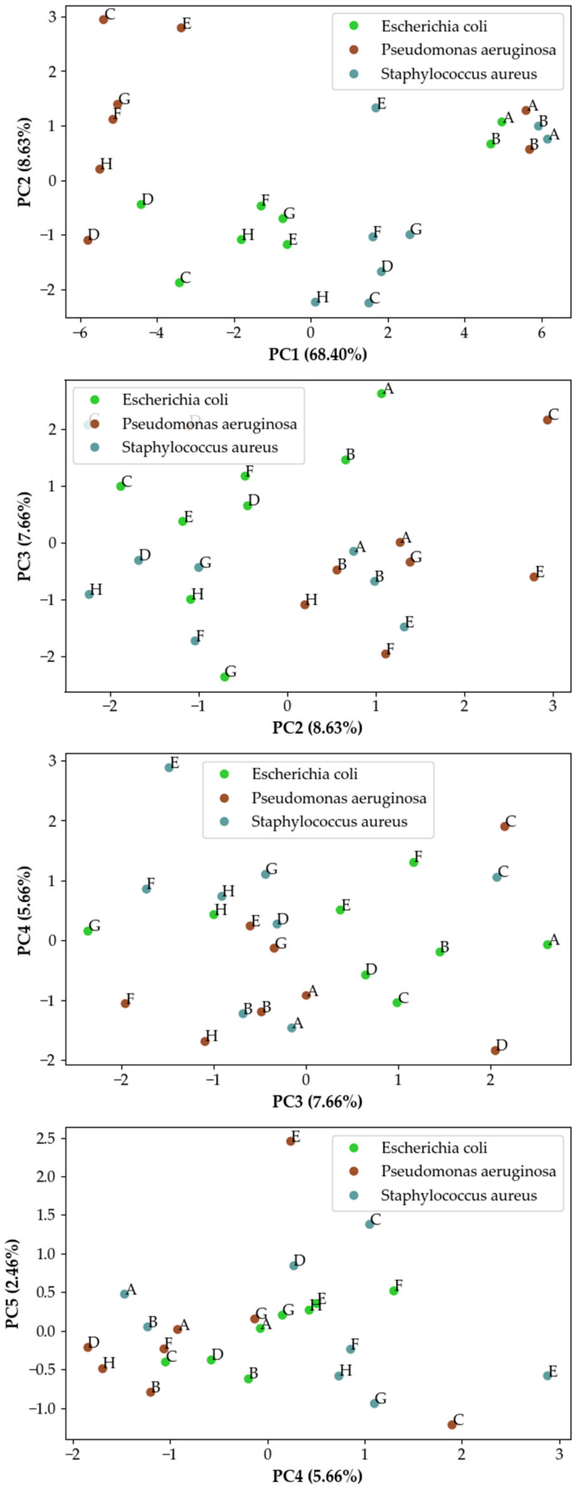

Figure S6b. PCA representation of the multi-wavelength measurements for SAg/AuNPs-WT

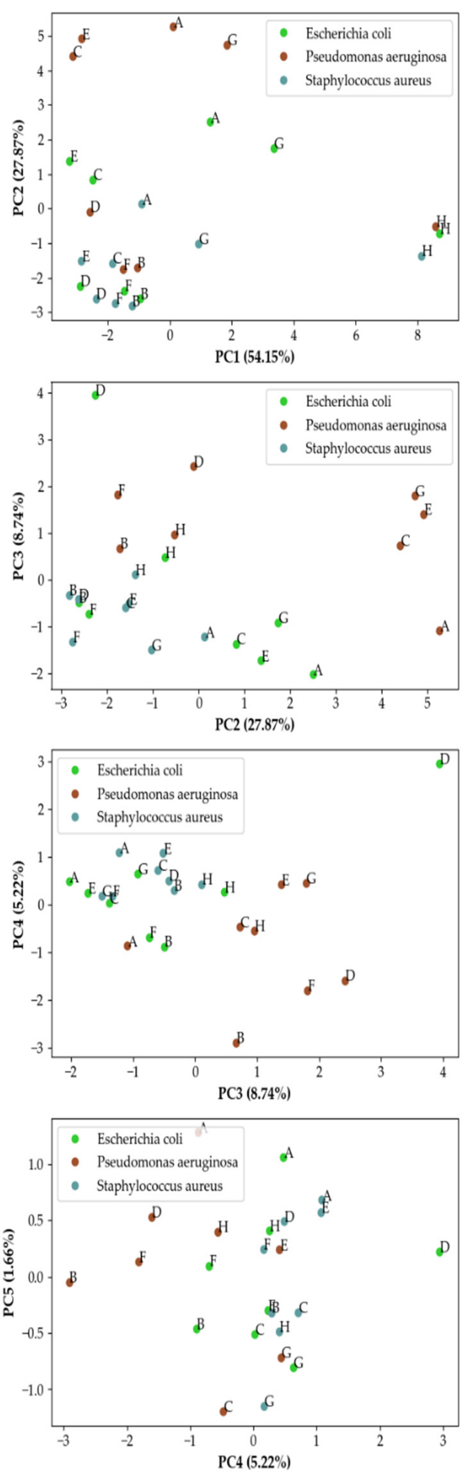

Figure S7a. PCA representation for the single wavelength measurements for hydrogels.

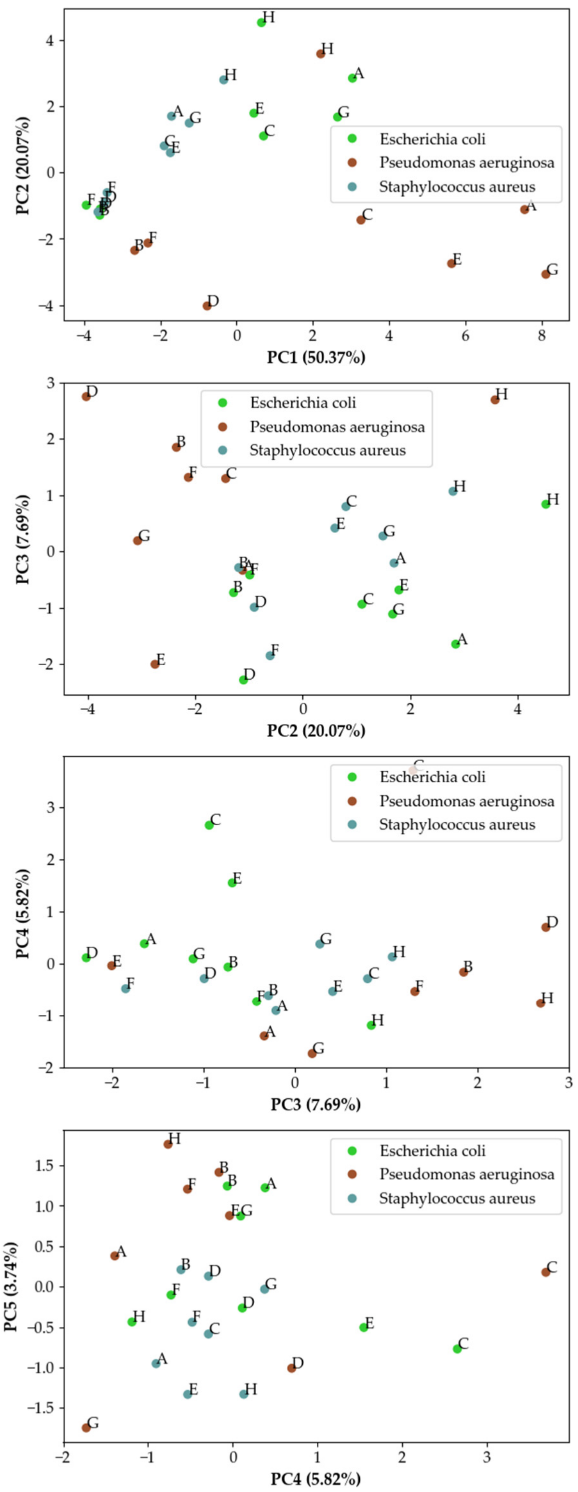

Figure S7b. PCA representation of the multi-wavelength measurements for hydrogels.

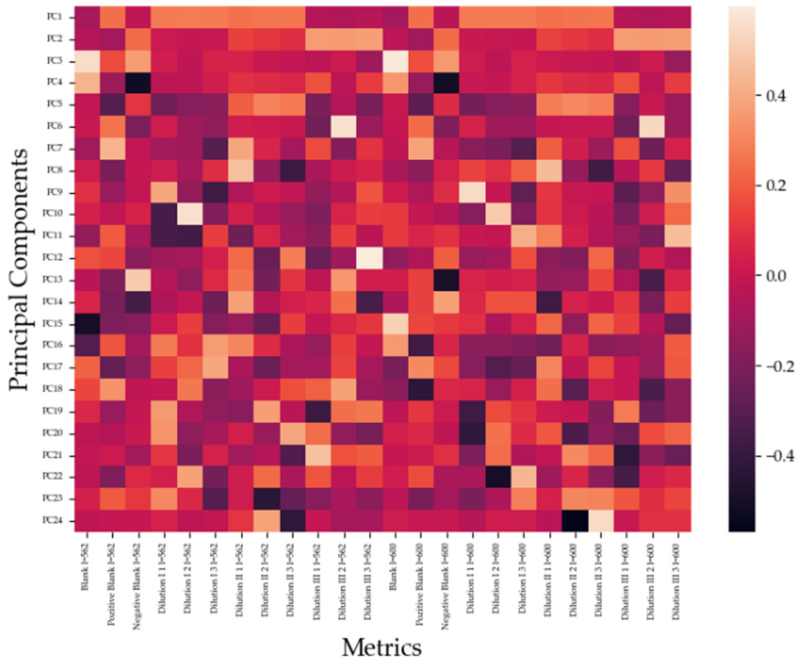

Figure S8a. Feature map for the single wavelength measurements for hydrogels.

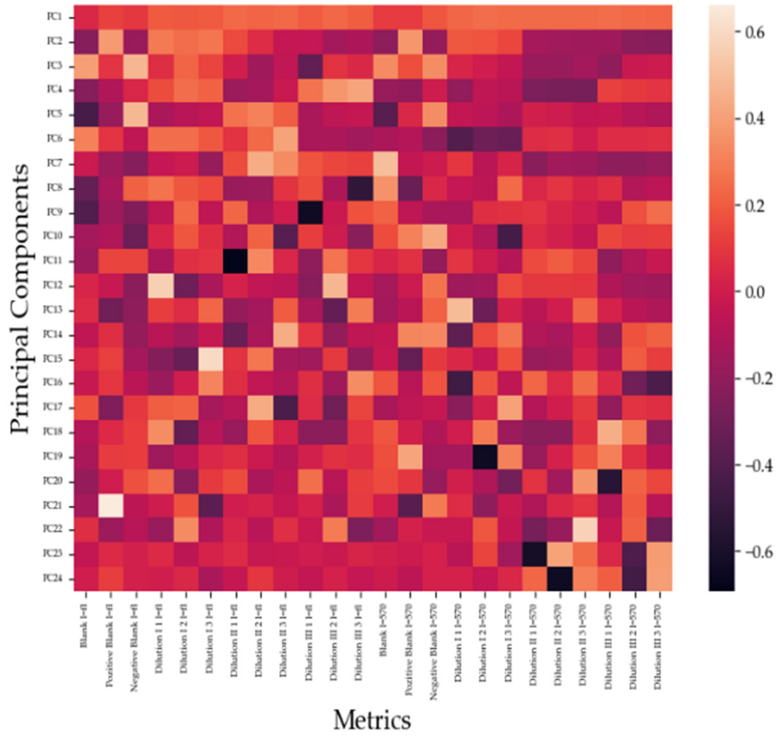

Figure S8b. Feature map for the multi-wavelength measurements for hydrogels.

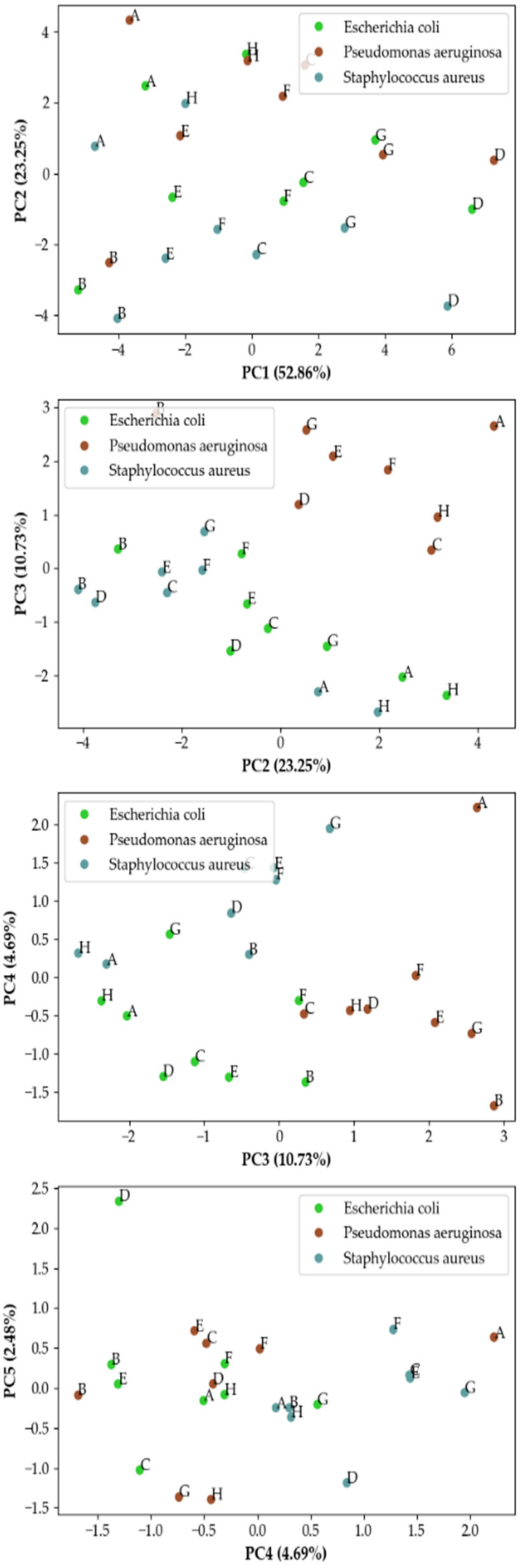

Figure S9a. PCA representation for the single wavelength measurements for ointments.

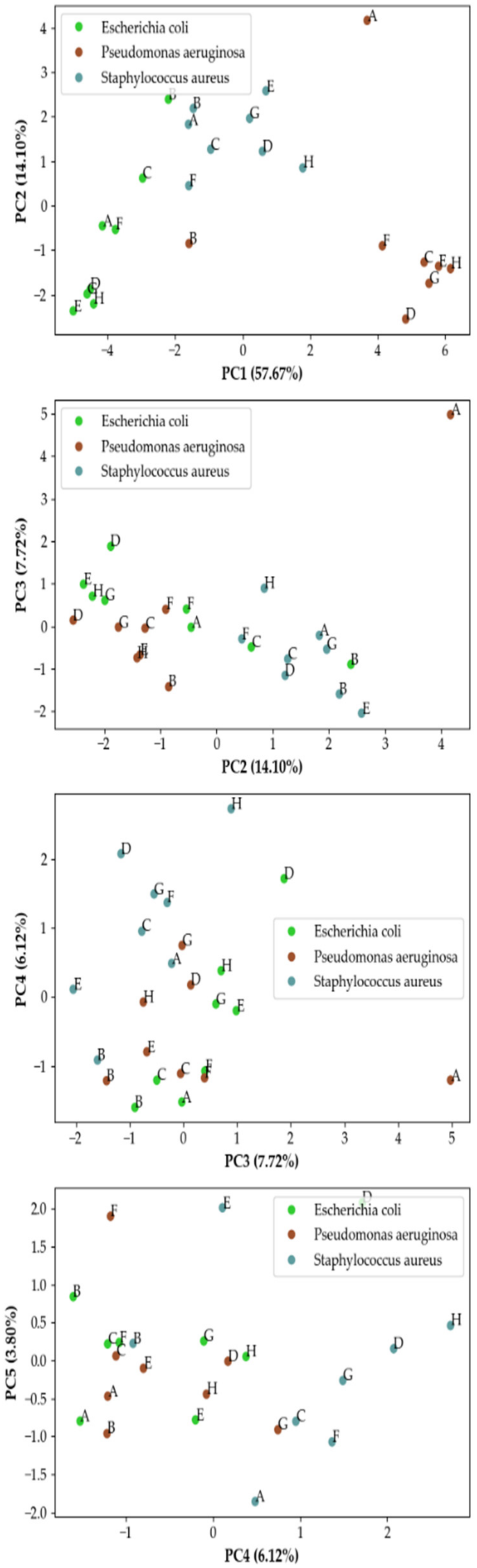

Figure S9b. PCA representation of the multi-wavelength measurements for ointments

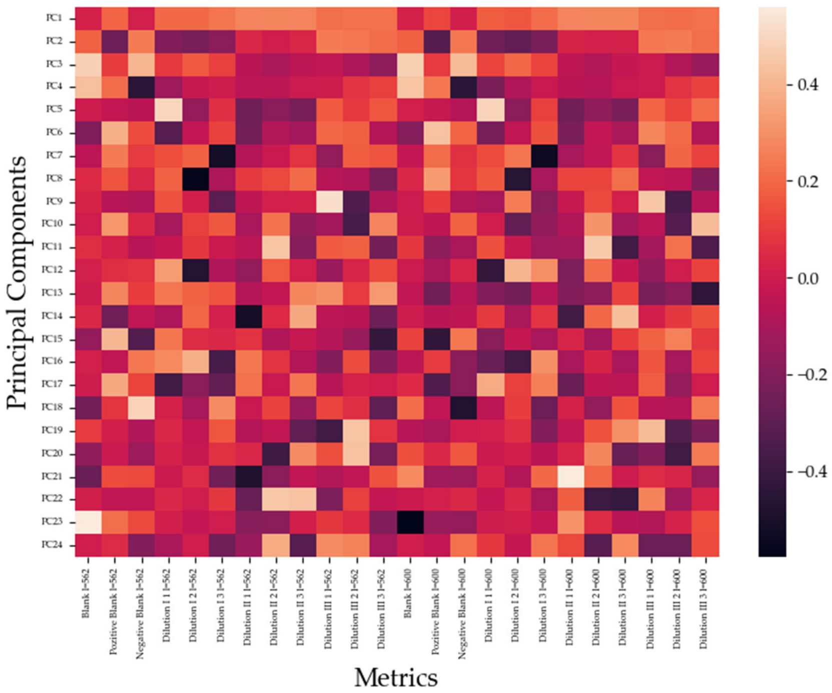

Figure S10a. Feature map for the single wavelength measurements for ointments.

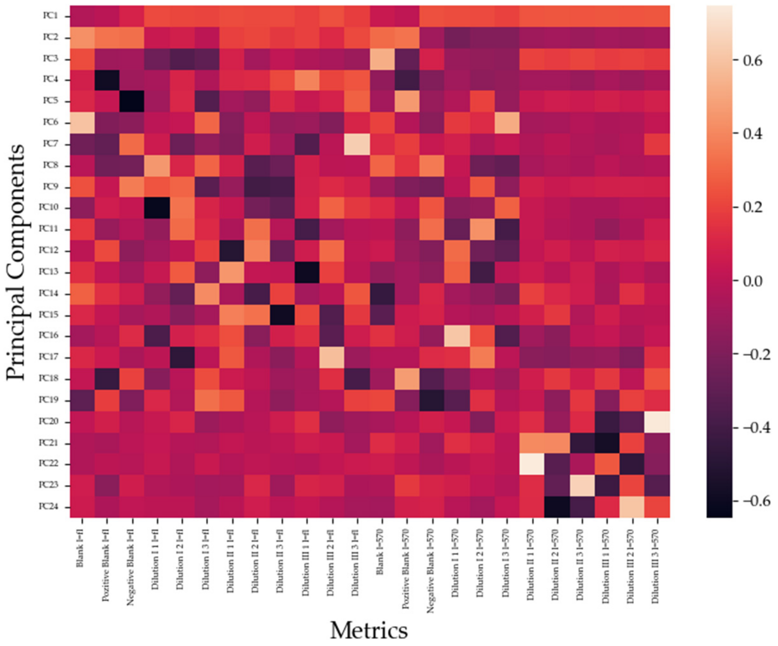

Figure S10b. Feature map for the multi-wavelength measurements for ointments.

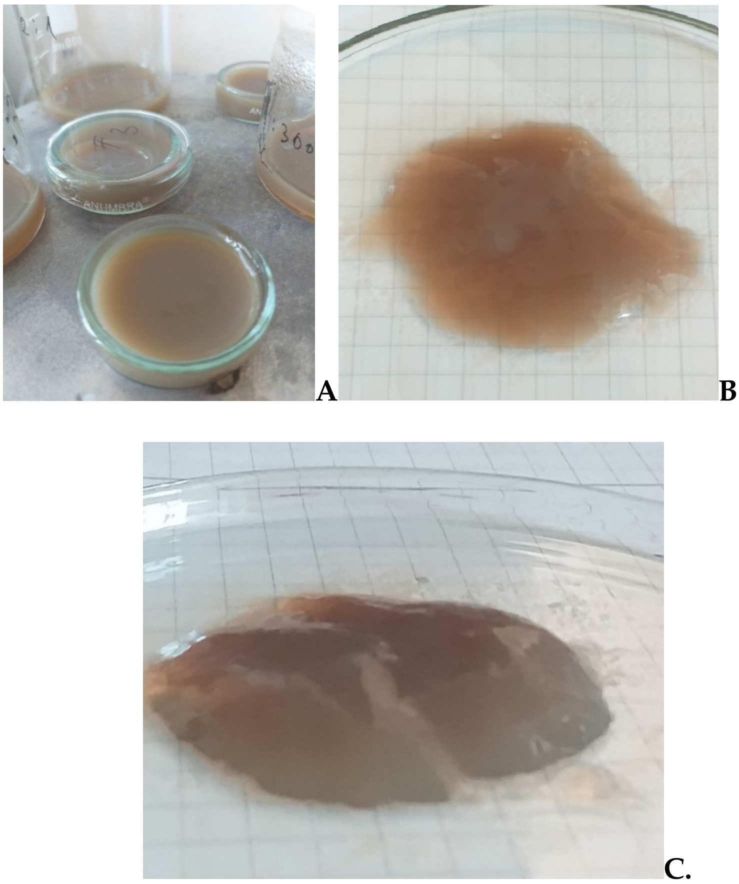

Figure S11. Hydrogels with: A. chitosan-gelatin-SAgNPs-WT volume ratio = 3:7 and 5 mL SAgNPs-WT; B. ratio = 5:5 (v/v) ; C. ratio= 1:9
